# Gut- and oral-dysbiosis differentially impact spinal- and bulbar-onset ALS, predicting ALS severity and potentially determining the location of disease onset

**DOI:** 10.1186/s12883-022-02586-5

**Published:** 2022-02-21

**Authors:** Harper S. Kim, John Son, Donghwan Lee, Joy Tsai, Danny Wang, E. Sandra Chocron, Seongwoo Jeong, Pamela Kittrell, Charles F. Murchison, Richard E. Kennedy, Alejandro Tobon, Carlayne E. Jackson, Andrew M. Pickering

**Affiliations:** 1 Center for Neurodegeneration and Experimental Therapeutics, Department of Neurology, University of Alabama at Birmingham, Birmingham, AL USA; 2 Medical Scientist Training Program, University of Alabama at Birmingham, Birmingham, AL USA; 3 Barshop Institute for Longevity and Aging Studies, University of Texas Health San Antonio, San Antonio, TX USA; 4 Medical Scientist Training Program, University of Texas Health San Antonio, San Antonio, TX USA; 5 Department of Anesthesiology, University of California Irvine, Irvine, CA USA; 6 School of Medicine, University of Texas Health San Antonio, San Antonio, TX USA; 7 Department of Anesthesiology, Massachusetts General Hospital, Boston, MA USA; 8 Department of Anesthesiology, Stanford University, Stanford, CA USA; 9 School of Nursing, University of Alabama at Birmingham, Birmingham, AL USA; 10 Department of Molecular Medicine, University of Texas Health San Antonio, San Antonio, TX USA; 11 Department of Neurology, University of Texas Health San Antonio, San Antonio, TX USA; 12 Department of Neurology, South Texas Veteran Health Care System, San Antonio, TX USA

**Keywords:** Amyotrophic lateral sclerosis, Microbiome, Lou Gehrig’s disease

## Abstract

**Background:**

Prior studies on the role of gut-microbiome in Amyotrophic Lateral Sclerosis (ALS) pathogenesis have yielded conflicting results. We hypothesized that gut- and oral-microbiome may differentially impact two clinically-distinct ALS subtypes (spinal-onset ALS (sALS) vs. bulbar-onset ALS (bALS), driving disagreement in the field.

**Methods:**

ALS patients diagnosed within 12 months and their spouses as healthy controls (*n* = 150 couples) were screened. For eligible sALS and bALS patients (*n* = 36) and healthy controls (*n* = 20), 16S rRNA next-generation sequencing was done in fecal and saliva samples after DNA extractions to examine gut- and oral-microbiome differences. Microbial translocation to blood was measured by blood lipopolysaccharide-binding protein (LBP) and 16S rDNA levels. ALS severity was assessed by Revised ALS Functional Rating Scale (ALSFRS-R).

**Results:**

sALS patients manifested significant gut-dysbiosis, primarily driven by increased fecal *Firmicutes/Bacteroidetes-*ratio (*F/B-*ratio). In contrast, bALS patients displayed significant oral-dysbiosis, primarily driven by decreased oral *F/B-*ratio. For sALS patients, gut-dysbiosis (a shift in fecal *F/B-*ratio), but not oral-dysbiosis, was strongly associated with greater microbial translocation to blood (*r* = 0.8006, *P* < 0.0001) and more severe symptoms (*r* = 0.9470, *P* < 0.0001). In contrast, for bALS patients, oral-dysbiosis (a shift in oral *F/B-*ratio), but not gut-dysbiosis, was strongly associated with greater microbial translocation to blood (*r* = 0.9860, *P* < 0.0001) and greater disease severity (*r* = 0.9842, *P* < 0.0001). For both ALS subtypes, greater microbial translocation was associated with more severe symptoms (sALS: *r* = 0.7924, *P* < 0.0001; bALS: *r* = 0.7496, *P* = 0.0067). Importantly, both sALS and bALS patients displayed comparable oral-motor deficits with associations between oral-dysbiosis and severity of oral-motor deficits in bALS but not sALS. This suggests that oral-dysbiosis is not simply caused by oral/bulbar/respiratory symptoms but represents a pathological driver of bALS.

**Conclusions:**

We found increasing gut-dysbiosis with worsening symptoms in sALS patients and increasing oral-dysbiosis with worsening symptoms in bALS patients. Our findings support distinct microbial mechanisms underlying two ALS subtypes, which have been previously grouped together as a single disease. Our study suggests correcting gut-dysbiosis as a therapeutic strategy for sALS patients and correcting oral-dysbiosis as a therapeutic strategy for bALS patients.

**Supplementary Information:**

The online version contains supplementary material available at 10.1186/s12883-022-02586-5.

## Introduction

**Fig. 1:**
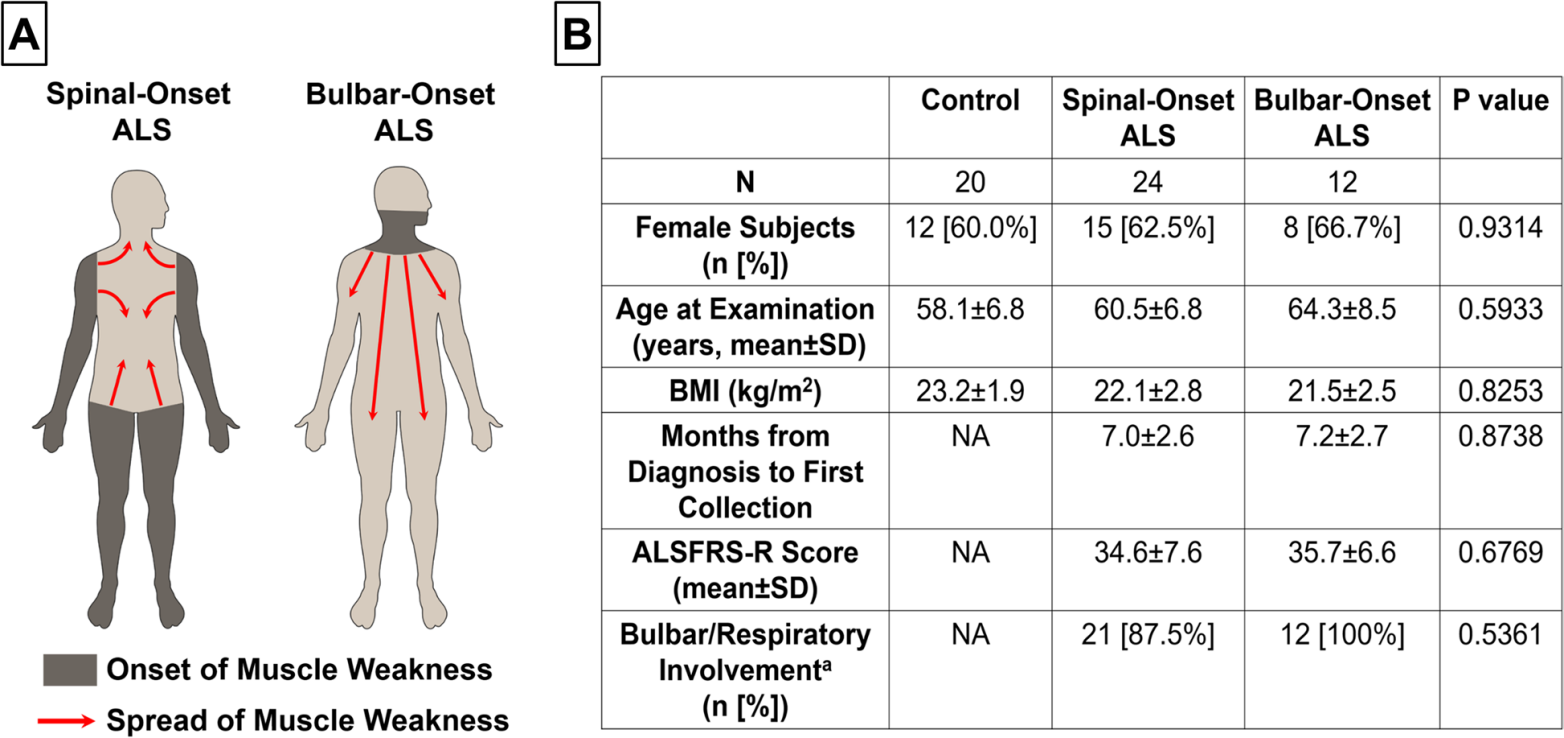
Demographic and Clinical Characteristics of the Cohort. **A** Pattern of motor involvement in two clinically-distinct ALS subtypes. In spinal-onset ALS, the muscle weakness starts in limbs but radiates to other body regions including head and neck. In bulbar-onset ALS, the muscle weakness starts in head and neck but radiates to other body regions including limbs. **B** Demographic and clinical characteristics of healthy controls, spinal-onset ALS patients, and bulbar-onset ALS patients. ^a^Bulbar/Respiratory involvement was defined as a score ≤ 20 on bulbar and respiratory items of the ALSFRS-R [7]. Abbreviation: ALSFRS-R; Revised ALS Functional Rating Scale, BMI; Body Mass Index. Statistics: For comparisons of age, BMI, months from diagnosis to first collection, and ALSFRS-R score, Kruskal–Wallis test with Dunn’s post-hoc multiple comparisons test was utilized to compute *P* values. For comparisons of sex and bulbar/respiratory involvement, Fisher’s exact test was utilized to compute *P* values

Amyotrophic lateral sclerosis (ALS) is a phenotypically heterogeneous neurodegenerative disorder with survival ranging from a few months to over 20 years [1]. This substantial heterogeneity has contributed to poor clinical trial outcomes and a lack of effective treatments [1]. A reliable predictor of ALS progression is where paralysis first starts before spreading to other body regions [1–4] (Fig. 1A). Bulbar-onset ALS (bALS) patients (20 ~ 25% of cases), whose muscle weakness initiates in the head and neck, have the worst prognosis (10-year survival rate: 3.4%), whereas spinal-onset ALS (sALS) patients (75 ~ 80% of cases), whose symptoms initiate in the limbs, show a much better prognosis (10-year survival rate: 13.0%) [1–4]. Additionally, bALS patients have nearly twice as fast disease progression, much shorter survival, and 4.5-fold higher mortality risk compared to sALS patients [1–4]. Consistent with distinct clinical features, these two ALS subtypes manifest different pathological signatures [5], impact different brain regions [6], and are associated with different sets of genetic risk factors [1].

Despite clear clinico-pathological differences, sALS and bALS are considered the same disease. This represents a critical problem since sALS and bALS patients often respond differently to treatments [8, 9]. Many drugs are effective in only one subtype [8, 9]. For example, riluzole, the first FDA-approved drug available to treat ALS, improved median survival by 2.8-fold in bALS patients (7.5-months vs. 18-months) but conferred no survival advantage in sALS patients (18-months vs. 18-months) [9]. The molecular mechanisms that differentiate between sALS and bALS are unknown, and there are no established animal models for bALS [10]. Thus, clinical research to identify the factors that determine the location of disease onset in ALS is crucial. This will enable researchers and clinicians to develop therapy targeting specific ALS subtypes and thereby more effectively treat ALS.

Microbial-dysbiosis (imbalance in microbial composition) has begun to emerge as an important disease modifying factor in neurodegenerative disorders such as Alzheimer’s disease (AD) and Parkinson’s disease (PD). Dysbiosis is reported to weaken mucosal barriers, enabling microbes to translocate to the blood [11] and alter multiple endocrine, immune, metabolic, and neural pathways, all of which may contribute to the pathogenesis of neurodegenerative disorders [12, 13]. Gut-dysbiosis, evident in AD and PD, can trigger Aβ-plaque depositions and α-synuclein aggregation and cause neurodegeneration [14–18]. Restoring normal gut microbiota and correcting gut-dysbiosis via fecal transplantation, antibiotics, or probiotics ameliorated AD/PD pathologies in animal models [16, 19–21]. Additionally, preventing gut-dysbiosis in AD/PD animal models by housing them in a germ-free environment drastically reduced Aβ-plaques and α-synuclein inclusions [14, 18].

Not only gut-dysbiosis but also oral-dysbiosis has recently been suggested to play central roles in AD and PD pathogenesis. For example, in PD patients, oral microbiome changes were highly predictive of their impairments in locomotion, balance, and cognition [22]. Studies with post-mortem brain tissues from AD patients and animal models suggest that several periodontal pathogens are translocated to the specific brain regions [23, 24], potentially driving lacunar infarctions [25], the commonest vascular cause of dementia. Additionally, significantly elevated levels of IgG against specific periodontal bacteria (e.g. *Porphyromonas gingivalis*) were highly predictive of cognitive/memory impairments in older adults and patients with AD and dementia [26–30]. Further, chronic application of periodontal pathogens significantly increased Aβ productions and led to brain inflammation and neurodegeneration in wild-type mice [31]. Orally bioavailable, brain penetrant inhibitor against Kgp, the essential component for pathogenicity of *Porphyromonas gingivalis*, is currently being tested in human clinical studies for AD [32].

Based on animal studies, microbial-dysbiosis appears to play a significant role in ALS pathogenesis as well. Rodent models of ALS displayed gut-dysbiosis before the onset of clinical symptoms, which damaged intestinal tight-junctions and increased gut permeability [33–35]. Drastic changes in specific gut-microbial species and subsequent gut-dysbiosis were highly correlated with ALS disease severity [33]. Correcting gut-dysbiosis slowed down ALS progression, whereas exacerbating gut-dysbiosis accelerated disease progression in animal models [33, 35].

However, microbiome studies with ALS patients have yielded conflicting results. Some studies report a correlation between gut-microbiota and ALS susceptibility/severity [33, 36], while others report no association [37]. Interestingly, most microbiome studies with ALS patients have not distinguished between ALS subtypes, and many of these studies vary in relation to inclusion of sALS and bALS patients [33, 36, 37]. Though, a recent study with 2 bALS and 17 sALS patients has reported distinct fecal cytokine profiles in two ALS subtypes [38]. Additionally, all the animal studies that yielded positive correlations between gut-dysbiosis and ALS severity exclusively used sALS animal models [10, 33–35]. We thus hypothesized that microbial differences between the two ALS subtypes may represent a confounding factor driving disagreement in the field. Further, we postulated that unique microbiome signatures in different organs (gut vs. oral cavity) can differentiate between sALS and bALS and determine the location of disease onset. To test these hypotheses, we examined gut- and oral-microbiome changes in ALS patients stratified by locations of disease onset.

## Methods

### Study enrollment and patient assessment

All participants provided written informed consent, and all the methods were conducted according to the IRB-approved protocol 20170646HU. Patients diagnosed with probable/definite ALS within 12 months according to the revised El-Escorial criteria were recruited from the South Texas ALS Clinic. Patients using non-invasive ventilation and/or feeding tubes (PEG) or diagnosed with other psychiatric/neurological disorders were excluded. Patients were classified based on location of disease onset (spinal-/bulbar-onset). Spouses of ALS patients were recruited as healthy controls if they did not show any early signs of ALS/other neurological disorders and if they did not have a 1^st^-degree relative or more than one relative with ALS. Exclusion criteria for patients and controls included exposure to antibiotics/probiotics, immunocompromising illness/therapy, previous abdominal/anorectal surgery, GI-/respiratory-/gynecological-tract infection, food poisoning, or major epistaxis requiring treatment, active/persistent primary disease of GI-/respiratory-/gynecological-tract, endocrinal disease, heart failure, severe renal-insufficiency, current pregnancy, drug/alcohol abuse, and active smoking within 6-months. At the time of sample collections, ALS severity was assessed by Revised ALS Functional Rating Scale (ALSFRS-R) that evaluates the functional status of patients ranging from 0 (worst function) to 48 (best function) [7].

### ALSFRS-R functional assessment

ALS symptom severity is assessed by the ALSFRS-R score (12 item assessment); the assessment can be divided into 4 domains (Bulbar, Fine Motor, Gross Motor, and Respiratory). The Bulbar domain includes (1) speech, (2) salivation, and (3) swallowing. The Fine Motor subscore includes (1) handwriting, (2) cutting food and handling utensils, and (3) dressing and hygiene. The Gross Motor score includes (1) turning in bad and adjusting bed clothes, (2) walking, and (3) climbing stairs. The Respiratory subscore includes (1) dyspnea, (2) orthopnea, and (3) respiratory insufficiency. In each category, patients are scored between 4-no deficits to 0-signifcant deficits or unable to perform task. Individual item scores are summed to produce a reported score ranging from 0 (worst function) to 48 (best function). The ALSFRS-R score has been heavily validated and is the primary instrument used to assess ALS severity in the clinic [39].

### Sample collection and DNA extraction

During the clinic visit, the subjects spit approximately 5 mL of saliva into tubes pre-filled with saliva DNA stabilizer (Norgen Saliva DNA Collection and Preservation Devices). Additionally, approximately 10 mL of venous blood was drawn and collected with QIAamp DNA Blood Mini Kit. Then, each subject took home a stool sample collection kit (Fisherbrand Commode Specimen Collection System) along with instructions for collecting/mailing the specimen, exam gloves, alcohol wipes, and a postage-paid return mailing box filled with dry ice. Subjects fast overnight, collected their stool samples at home in the next morning, and immediately placed them on dry ice. Immediately after collection, participants mailed the samples directly to the lab so that samples could be received the next day. Upon the receipt of samples, they were stored at -80 °C after being checked for sample adequacy. Fecal samples were lysed by bead beating, and DNA was extracted using QIAamp DNA Stool Mini Kit, following manufacturer’s instructions. DNA from saliva samples was extracted with Norgen Saliva DNA Isolation Kit, following manufacturer’s instructions.

### 16S rRNA next-generation sequencing (NGS) and qPCR

Gut- and oral-microbial diversity was assessed by deep sequencing the V4 hypervariable region of bacterial 16S rRNA. Library preparation was performed by SeqMatic facility (Fremont, CA) per Illumina 16S metagenomics-sequencing library preparation protocol [40]. Sequencing was performed by SeqMatic via Illumina MiSeq and sequences were aligned to reference genomes. Illumina BaseSpace software was used for data analysis. For the major bacteria that were identified to be altered in 16S rRNA NGS (*Firmicutes, Bacteroidetes,* and *Fusobacteria*), SYBR qPCR (Roche LightCycler480 Real Time PCR) was performed using the primer sets described in previous studies [41, 42]. All reads were assessed by read quality score. Any read with a score below Q_20_ was filtered out. The proportion of reads which met our minimum quality requirements is shown in Supplemental Fig. 19. The percent of reads which could be linked to a known taxon is also shown in Supplemental Fig. 19.

### Quantifying microbial translocation to blood

Plasma lipopolysaccharide-binding protein (LBP) levels were determined by the Human LBP ELISA kit (R&D Systems), following manufacturer’s instructions. DNA was extracted from blood plasma via Qiagen DNeasy Blood & Tissue Kits, following the manufacturer’s protocol. Then, total bacterial 16S rDNA in plasma was quantified via SYBR qPCR (Roche LightCycler480 Real Time PCR) using the primer sets and protocol described in the previous study [43].

### Oral health assessment

Salivary pH was digitally measured through a digital pH sensor (Fisher Scientific Accumet AE150). We initially calibrated the device using buffered solutions with pH 4.0, pH 7.0, and pH 10.0. After calibration, the sensor probe was dipped in saliva filled tube, where it remained for 30 s, thus yielding automatic pH reading. High sensitivity enzyme-linked immunosorbent sandwich assay kit (ELISA kit; Lifespan Biosciences LS-F27082) was used to determine the MUC7 levels in the saliva samples, following the protocol described by the manufacturer. Values of absorbance were read at the wavelength of A = 450 nm using the SpectraMax iD3 Multi-Mode Microplate Reader. Salivary MUC7 levels were estimated from standard curves, derived from the recombinant MUC7 standards. DNA copies of *Streptococcus Mutans*, *Lactobacillus spp*, and *Candida Albcians* were quantified via SYBR qPCR (Roche LightCycler480 Real Time PCR), using the primer sets and protocol described in previous studies [44, 45].

### Statistics

Analyses were performed with GraphPad Prism 8.0. Principal Component Analysis was conducted using the *prcomp* command from the *stats* package in RStudio. Microbiome cluster analysis at the phylum level was done using the Bray dissimilarity measure. Based on D’Agostino & Pearson test and Shapiro–Wilk test, some of our data showed non-normal distribution. We thus decided to use non-parametric tests for our data analysis. To be specific, in experiments comparing controls and ALS patients (combined), Mann–Whitney test was used. For analyses comparing controls, spinal-onset ALS patients, and bulbar-onset ALS patients, Kruskal–Wallis test with Dunn’s post-hoc test was used. For categorical variables, Fisher’s exact test was performed. For determining statistical significance of correlations, Spearman’s correlation coefficient (*r*) test was conducted. OTU richness measures the count of different species represented in a community. Shannon index measures the number of OTUs in the sample (richness) but scales them based on the *evenness* of the community. OTU richness and Shannon index were calculated using the vegan package in R [46]. OTU richness was calculated as the sum of the present OTUs. Shannon diversity was calculated as the exponential function of Shannon entropy. For all statistical analyses, a 2-sided *P* < 0.05 was accepted as statistically significant. All analyses were adjusted for multiple comparisons. We have included post-hoc power analyses demonstrating our study to be sufficiently powered (Supplemental Fig. 20).

## Results

### Demographic and clinical characteristics of the cohort

ALS is a rare and rapidly progressive disease, posing substantial challenges for patient recruitment. Total of 150 ALS patients diagnosed within 12-months were screened. To minimize the influence of lifestyle/socioeconomic factors (including diets) on microbiota [47], patient spouses were used as healthy controls. After screening, 36 ALS patients met the rigorous eligibility criteria (see Methods). To examine if gut- and oral-microbiome can differentiate between spinal-onset ALS (sALS) and bulbar-onset ALS (bALS), fecal and saliva samples were collected from 24 sALS patients and 12 bALS patients. Demographic and clinical features are summarized in Fig. 1B. ≈60–67% of participants were females. Controls were 58.1 ± 6.8 years-old, sALS patients were 60.5 ± 6.8 years-old, and bALS patients were 64.3 ± 8.5 years-old (no significant differences in sex/age distribution). Additionally, no significant differences between groups were observed in distributions of body mass index (BMI) and disease duration. Revised ALS Functional Rating Scale (ALSFRS-R) scores (see Methods) were 34.6 ± 7.6 for sALS patients and 35.7 ± 6.6 for bALS patients (no significant differences). At the time of sample collections, 87.5% of sALS patients showed clear oral/bulbar/respiratory symptoms (bulbar/respiratory ALSFRS-R subscore: 17.9 ± 2.9). This is consistent with previous reports that 85% of sALS patients display bulbar symptoms as the disease progresses and the paralysis spreads [5]. Since both sALS and bALS patients manifested oral/bulbar/respiratory symptoms (87.5% of sALS vs. 100% of bALS, *P* = 0.5361), oral/bulbar/respiratory symptoms should not be a confounder for oral-microbiome measures.

### Spinal-onset ALS patients manifest gut-dysbiosis, whereas bulbar-onset ALS patients display oral-dysbiosis

**Fig. 2:**
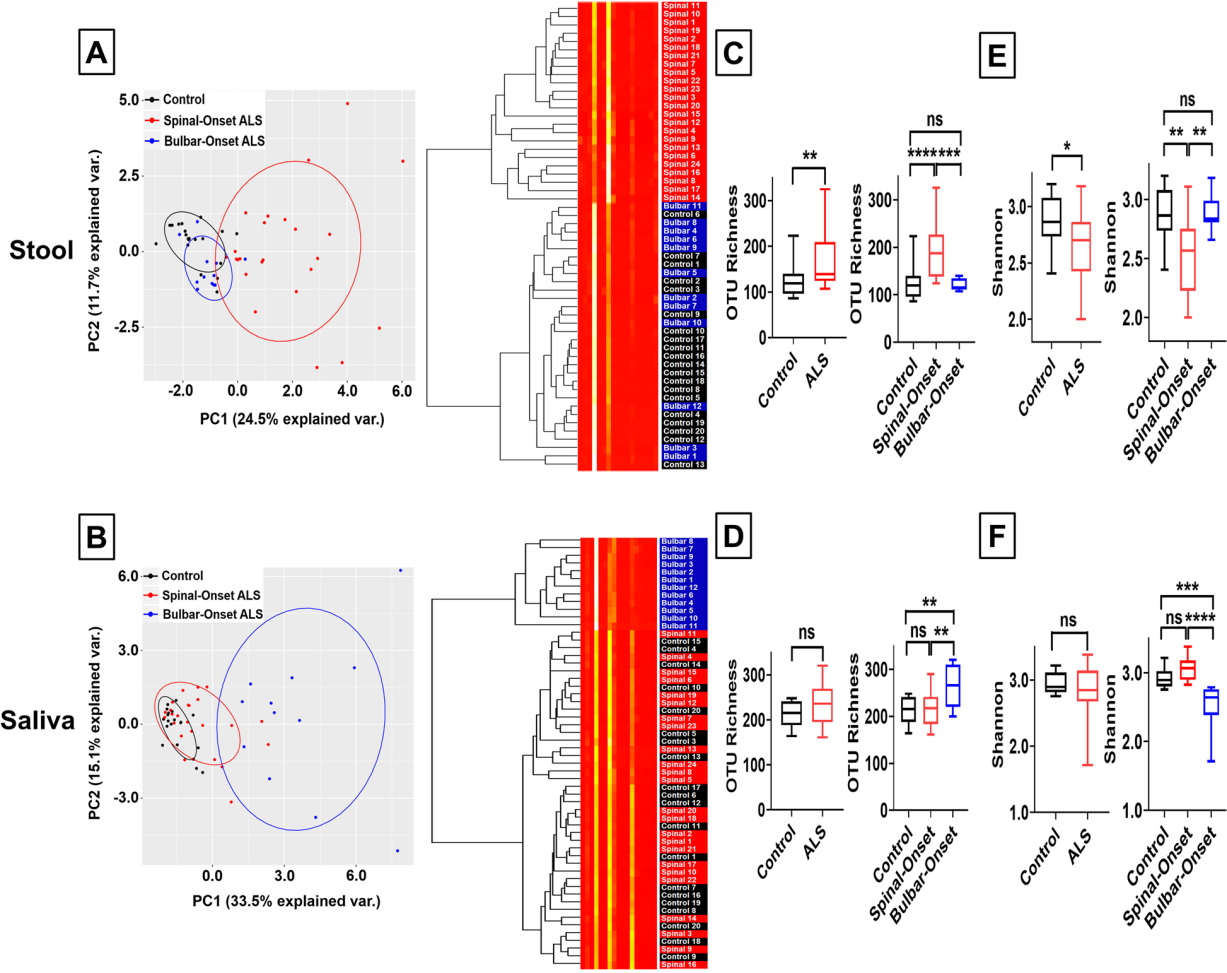
Spinal-Onset ALS Patients Manifest Gut-Dysbiosis, whereas Bulbar-Onset ALS Patients Display Oral-Dysbiosis. **A** Left panel represents principle component analysis of gut-microbiome species taxa at the phylum level. Right panel represents the heatmap for gut-microbiome distribution at the phylum level. Data derived from 20 healthy controls (black), 24 spinal-onset ALS patients (red), and 12 bulbar-onset ALS patients (blue). Spinal-onset ALS patients formed a distinct cluster from controls and bulbar-onset ALS patients. **B** Left panel represents principle component analysis of oral-microbiome species taxa at the phylum level. Right panel represents the heatmap for oral-microbiome distribution at the phylum level. Bulbar-onset ALS patients formed a distinct cluster from controls and spinal-onset ALS patients. **C** Gut-microbiome bacterial species number measured by Operational Taxonomic Unit (OTU) Richness. Results represent both ALS (spinal- and bulbar-onset ALS combined) or spinal- and bulbar-onset ALS quantified separately. Spinal-onset ALS patients displayed a significant increase in gut bacterial species count. No significant change in bulbar-onset ALS patients. **D** Oral-microbiome bacterial species number measured by OTU Richness. Bulbar-onset ALS patients displayed a significant increase in oral bacterial species count. No significant change in spinal-onset ALS patients. **E** Gut-microbiome species evenness measured through Shannon index. Spinal-onset ALS patients displayed a significant decrease in Shannon index, suggesting reduced gut-microbiome uniformity. No significant change in bulbar-onset ALS patients. **F** Oral-microbiome species evenness measured through Shannon index. Bulbar-onset ALS patients displayed a significant decrease in Shannon index, suggesting reduced oral-microbiome uniformity. No significant change in spinal-onset ALS patients. Statistics: For comparisons between controls and ALS patients (combined), Mann–Whitney test was utilized. For contrasts between control, spinal-onset ALS, and bulbar-onset ALS, Kruskal–Wallis test with Dunn’s post-hoc multiple comparisons test was utilized. Values represent sample median with interquartile range. Clustering was performed based on Bray Similarity matrix. *P < 0.05, **P < 0.01, ***P < 0.001, ****P < 0.0001

To compare the diversity and distribution of bacterial taxa between groups, the 5’ variable region (V4) of the bacterial 16S ribosomal RNA region was PCR amplified and subjected to next-generation sequencing. Next, Principle Component Analysis (PCA) and cluster analysis using the Bray dissimilarity measure were performed at the phylum level, with the latter summarized graphically using heatmaps. We found stool samples from sALS patients forming a distinct cluster from controls and bALS patients (Fig. 2A, Supplemental Fig. 1). Surprisingly, we observed completely opposite results in saliva samples, where bALS patients clustered separately from controls and sALS patients (Fig. 2B, Supplemental Fig. 2). These results suggest that gut-microbiome is altered in sALS, whereas oral-microbiome is altered in bALS.

To characterize how gut- and oral-microbiome are differentially affected in the two ALS subtypes, we calculated Operational Taxonomic Unit (OTU) richness, which quantifies the total number of observed bacterial species [48]. In stool samples, sALS patients had a significantly higher bacterial species count with no significant difference for bALS patients (Fig. 2C). Previous studies reported ALS patients overall to have significantly increased gut bacterial species number [36, 37]. Our results confirmed this, but the significance surprisingly arose solely from sALS patients (Fig. 2C). In contrast, for saliva samples, bALS patients had significantly greater bacterial species numbers with no significant difference for sALS patients (Fig. 2D).

We next measured Shannon-index, which reflects how evenly bacteria are distributed [48]. A decline in Shannon-index may indicate loss of bacterial diversity and dysbiosis [48]. In stool samples, sALS patients displayed a significantly lower Shannon-index (suggesting gut-dysbiosis) with no significant change in bALS patients (Fig. 2E). In contrast, in saliva, bALS patients exhibited a significantly lower Shannon-index (suggesting oral-dysbiosis) with no significant change in sALS patients (Fig. 2F). Since OTU richness informs on the richness of taxons while Shannon index conveys how evenly taxons are distributed within a population, our data suggest the increased numbers of total taxons with a small number of particular taxons unevenly dominating the stool (but not saliva) samples of sALS patients and the saliva (but not stool) samples of bALS patients. Thus, these results suggest that gut microbial-imbalance is prominent in sALS, while oral microbial-imbalance is prominent in bALS.

These analyses were each independently re-run directly comparing patients to matched household controls via matched-pair analysis as a means of accounting for environmental factors including diets. Household controls were the patient’s spouse or partner. The number of household controls is reduced in this analysis as some spouses or partners did not consent to participate in the study. However, the above conclusions were preserved in the matched-pair analysis (Supplemental Fig. 3A-D).

### Increased fecal *Firmicutes*/*Bacteroidetes* ratio drives gut-dysbiosis in spinal-onset ALS, whereas decreased oral *Firmicutes*/*Bacteroidetes* ratio drives oral-dysbiosis in bulbar-onset ALS

**Fig. 3:**
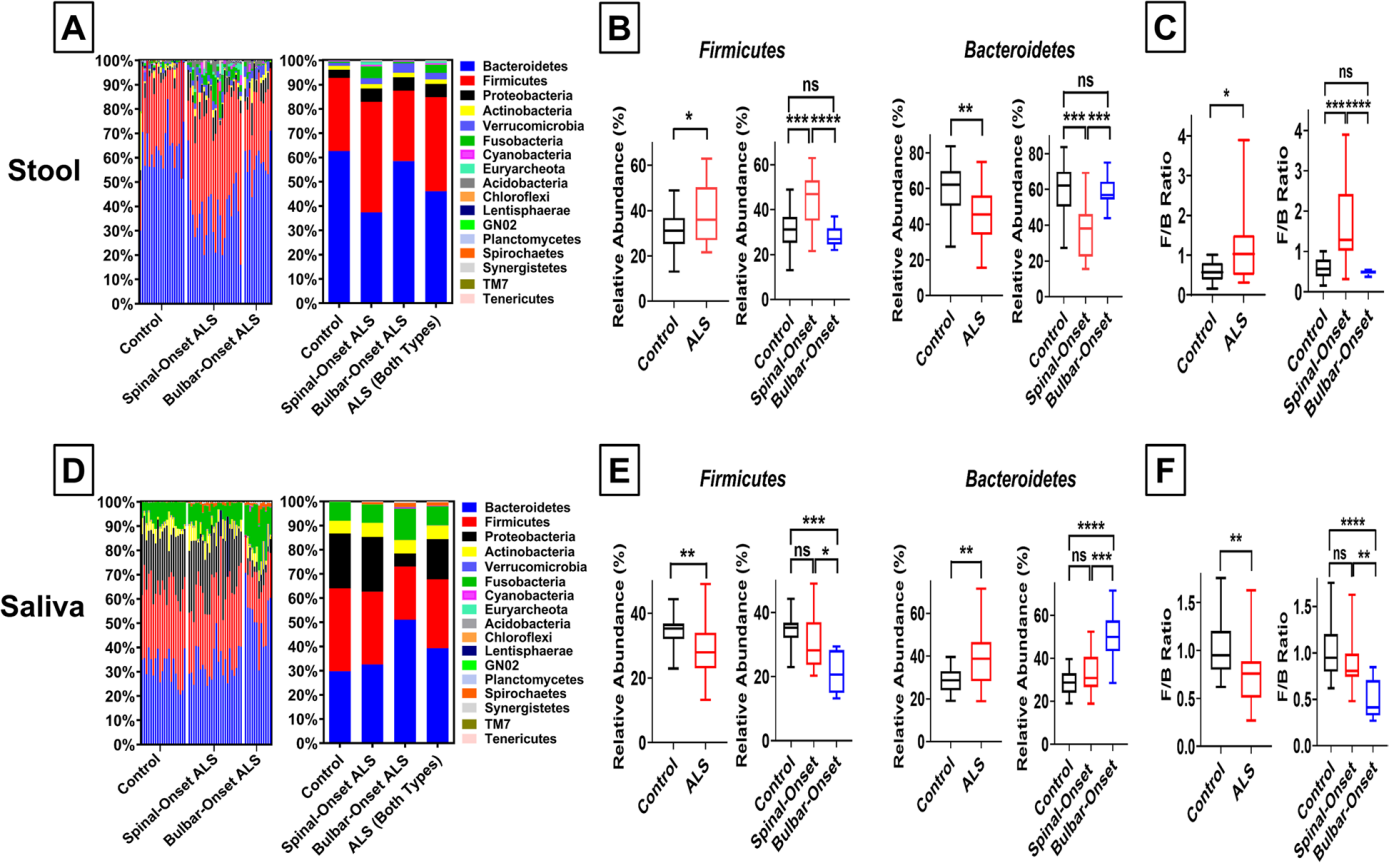
Increased Fecal *Firmicutes*/*Bacteroidetes* Ratio Drives Gut-Dysbiosis in Spinal-Onset ALS, whereas Decreased Oral *Firmicutes*/*Bacteroidetes* Ratio Drives Oral-Dysbiosis in Bulbar-Onset ALS. **A** Relative abundance of microbial phyla in gut microbiome comparing control, spinal-onset ALS patients alone, bulbar-onset ALS patients alone, and ALS patients (both spinal-and bulbar-onset ALS patients combined). Left panel represents taxonomic distribution for all the individuals; Right panel represents average taxonomic distribution for each group. **B** Percent abundance of *Firmicutes* and *Bacteroidetes* phyla in gut microbiome. Results represent both ALS (spinal- and bulbar-onset ALS combined) or spinal- and bulbar-onset ALS quantified separately. Spinal-onset ALS patients displayed a significant enrichment of *Firmicutes* and a significant depletion of *Bacteroidetes* in the gut. No significant change in bulbar-onset ALS. **C** Fecal ratio of *Firmicutes* to *Bacteroidetes* (*F*/*B*). Spinal-onset ALS patients displayed a significant increase in fecal *F*/*B* ratio. No significant change in bulbar-onset ALS. **D** Relative abundance of microbial phyla in oral microbiome. Left panel represents taxonomic distribution for all the individuals; Right panel represents average taxonomic distribution for each group. **E** Percent abundance of *Firmicutes* and *Bacteroidetes* phyla in oral microbiome. Bulbar-onset ALS patients displayed a significant depletion of *Firmicutes* and a significant enrichment of *Bacteroidetes* in the oral cavity. No significant change in spinal-onset ALS. **F** Oral *F*/*B* ratio. Bulbar-onset ALS patients displayed a significant decrease in oral *F*/*B* ratio. No significant change in spinal-onset ALS. Statistics: For comparisons between controls and ALS patients (combined), Mann–Whitney test was utilized. For contrasts between control, spinal-onset ALS, and bulbar-onset ALS, Kruskal–Wallis test with Dunn’s post-hoc multiple comparison test was utilized. Values represent sample median with interquartile range. *P < 0.05, **P < 0.01, ***P < 0.001, ****P < 0.0001

To identify the source of dysbiosis, we performed phylogenetic composition analysis (Fig. 3A, D). *Firmicutes* and *Bacteroidetes* comprise the majority of the gut- and oral-microbiome [49], and imbalance in the *Firmicutes*/*Bacteroidetes*-ratio (*F/B-*ratio) is associated with poor health outcomes [50]. In stool from controls, ≈30% of the microbiome was *Firmicutes* and ≈60% *Bacteroidetes* (≈0.5 *F*/*B*, Fig. 3A-C), consistent with other studies [49]. However, sALS patients, but not bALS patients, showed a significant enrichment of *Firmicutes* along with a depletion of *Bacteroidetes* in stool samples, resulting in a significant increase in fecal *F/B-*ratio (*F*/*B* > 1, Fig. 3A-C, Supplemental Figs. 3, 4). When both ALS subtypes were combined, patients still showed a significant increase in fecal *F*/*B-*ratio, but this was completely driven by changes in sALS patients with no significant changes in bALS patients (Fig. 3C). As with our other results, we observed a complete reversal in saliva samples. Healthy controls displayed ≈1/1 distribution of *Firmicutes* and *Bacteroidetes* (≈1 *F*/*B*, Fig. 3D-F). However, bALS patients, but not sALS patients, showed a significant depletion of *Firmicutes* and enrichment of *Bacteroidetes* (Fig. 3D, E, Supplemental Figs. 3, 4). This caused a significant decrease in oral *F/B-*ratio (*F*/*B* < 1) in bALS patients but not sALS patients (Fig. 3F). Together, these results suggest a shift in fecal *F/B-*ratio as the source for gut-dysbiosis in sALS and a shift in oral *F/B-*ratio as the source for oral-dysbiosis in bALS. As previously, these trends were preserved in matched-pair analyses (Supplemental Fig. 3E, F) and independently validated by qPCR (Supplemental Fig. 4).

**Fig. 4:**
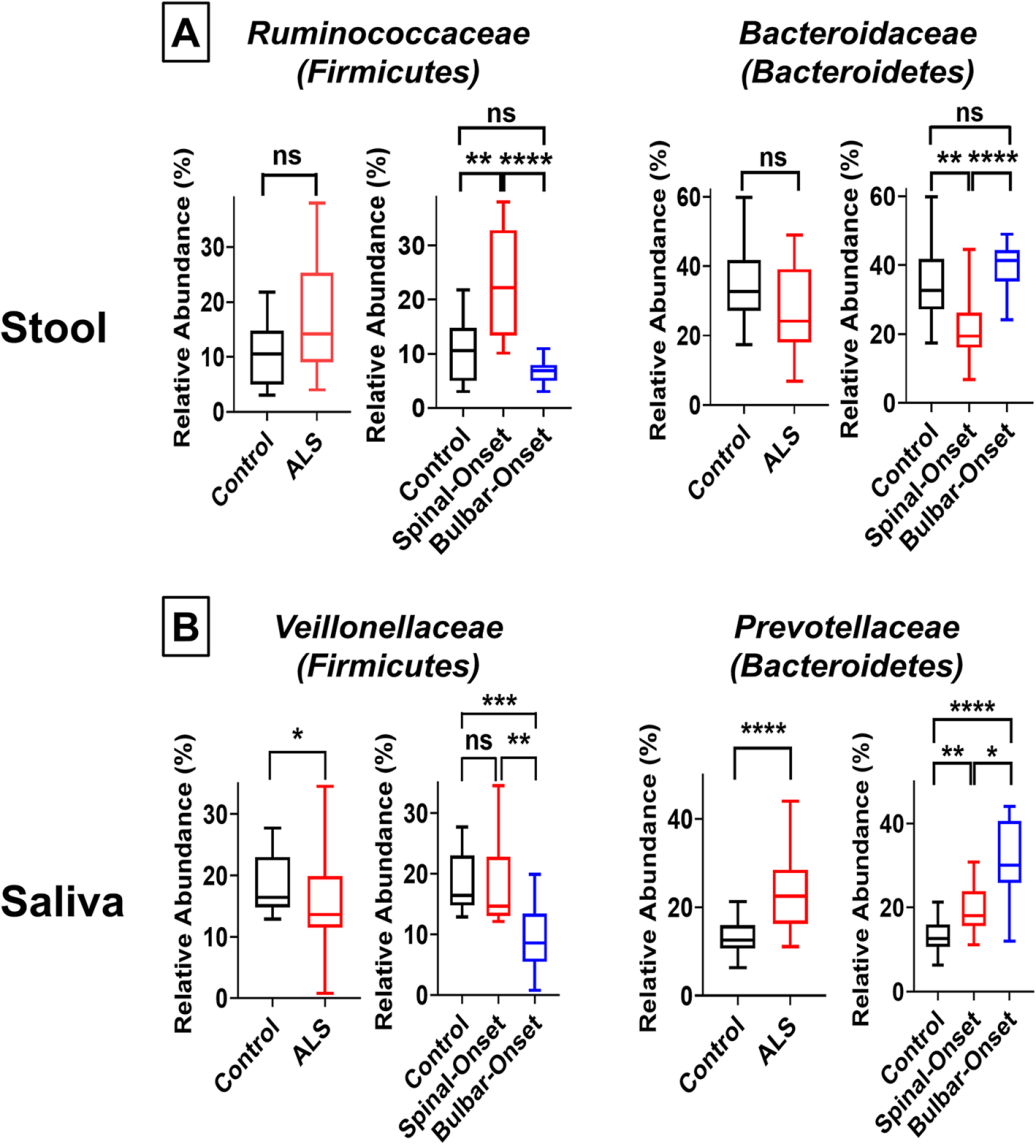
Major Bacterial Families Causing Increased Fecal *Firmicutes*/*Bacteroidetes* Ratio in Spinal-Onset ALS and Decreased Oral *Firmicutes*/*Bacteroidetes* Ratio in Bulbar-Onset ALS. **A** Percent abundance of *Ruminococcaceae* family (a member of the *Firmicutes* phyla) and *Bacteroidaceae* family (a member of the *Bacteroidetes* phyla) in the gut microbiome. Results represent both ALS (spinal- and bulbar-onset ALS combined) or spinal- and bulbar-onset ALS quantified separately. **B** Percent abundance of *Veilonellaceae* family (a member of the *Firmicutes* phyla) and *Prevotellaceae* family (a member of the *Bacteroidetes* phyla) in the oral microbiome. Statistics: For comparisons between controls and ALS patients (combined), Mann–Whitney test was utilized. For contrasts between control, spinal-onset ALS, and bulbar-onset ALS, Kruskal–Wallis test with Dunn’s post-hoc multiple comparisons test was utilized. Values represent sample median with interquartile range. *P < 0.05, **P < 0.01, ***P < 0.001, ****P < 0.0001

Because *F*/*B*-ratio represents a somewhat broad measure of dysbiosis, we performed a deeper investigation at the family level. Here, we found the shift in fecal *F/B-*ratio in sALS to be mainly driven by a significant enrichment of the *Ruminococcaceae* family (*Firmicutes* phyla) and depletion of the *Bacteroidaceae* family (*Bacteroidetes* phyla) (Fig. 4A). Similar to our results, AD/PD animal models and patients showed a twofold enrichment of *Ruminococcaceae* along with a 50% decrease in *Bacteroidaceae* in stool samples [51–53] and drastically increased fecal *F/B-*ratio [54]. The shift in oral *F/B-*ratio in bALS was mainly driven by a significant depletion of *Veillonellaceae* family (*Firmicutes* phyla) along with increased abundance of *Prevotellaceae* family (*Bacteroidetes* phyla) (Fig. 4B). Interestingly, PD patients also showed enrichment of *Prevotellaceae* in the oral cavity [55]. In addition, although the dysbiosis we observed was mainly driven by a shift in *F/B-*ratio, we also observed changes in other bacterial families/phyla (Supplemental Figs. 5–8). Some microbiota were affected in sALS or bALS only (Supplemental Figs. 5, 7) or both ALS subtypes (Supplemental Figs. 6, 8). Alterations in *Prevotellaceae* and *Proteobacteria* we observed in ALS (Supplemental Figs. 5–8) were previously reported in AD/PD as well and linked with disease severity [53, 56, 57].

### Gut-dysbiosis predicts disease severity of spinal-onset ALS, whereas oral-dysbiosis predicts disease severity of bulbar-onset ALS

**Fig. 5:**
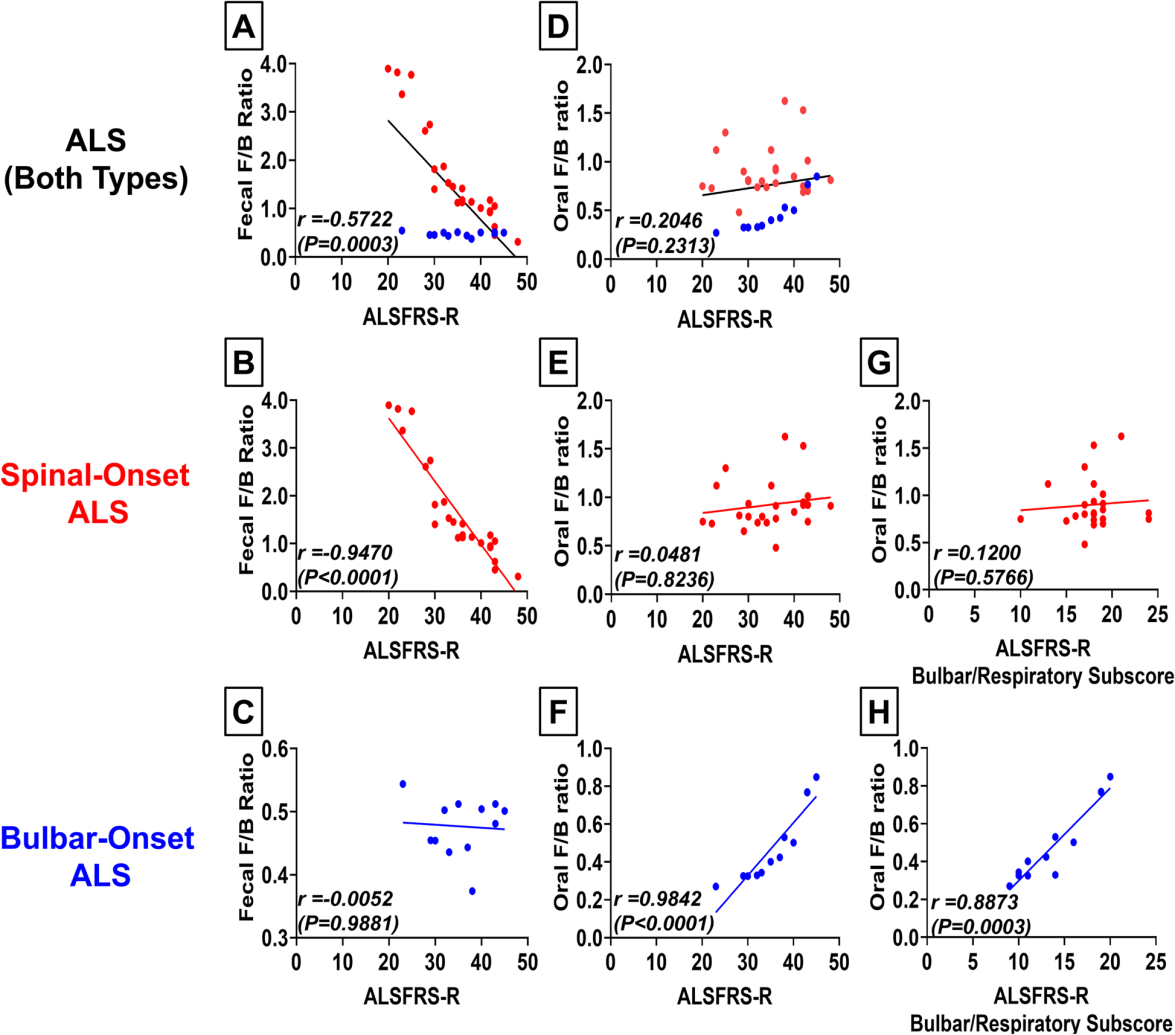
Gut-Dysbiosis Predicts Disease Severity of Spinal-Onset ALS, whereas Oral-Dysbiosis Predicts Disease Severity of Bulbar-Onset ALS. **A** Linear regression analysis comparing fecal *F*/*B* ratio and ALS severity score (ALSFRS-R) for all ALS patients combined. The ALSFRS-R gives a total of 48 points, and a lower ALSFRS-R score indicates greater motor impairment (0 = worst function; 48 = best function)[7]. Spinal-onset ALS patients shown in red, bulbar-onset ALS patients in blue. Higher fecal *F*/*B* ratio was strongly associated with lower ALSFRS-R score (greater ALS severity) with distinct patterns between the two ALS subtypes. **B** Linear regression analysis comparing fecal *F*/*B* ratio and ALSFRS-R score for spinal-onset ALS patients alone. In spinal-onset ALS patients, higher fecal *F*/*B* ratio was strongly associated with lower ALSFRS-R score (greater ALS severity). **C** Linear regression analysis comparing fecal *F*/*B* ratio and ALSFRS-R score for bulbar-onset ALS patients alone. Bulbar-onset ALS patients displayed no significant association between fecal *F*/*B* ratio and ALSFRS-R score/ALS severity. **D** Linear regression analysis comparing oral *F*/*B* ratio and ALSFRS-R score for all ALS patients combined. Spinal-onset ALS patients shown in red, bulbar-onset ALS patients in blue. We observed no overall trend for association between oral *F*/*B* ratio and ALSFRS-R score with distinct patterns between the two ALS subtypes. **E** Linear regression analysis comparing oral *F*/*B* ratio and ALSFRS-R score for spinal-onset ALS patients alone. Spinal-onset ALS patients displayed no significant association between oral *F*/*B* ratio and ALSFRS-R score/ALS severity. **F** Linear regression analysis comparing oral *F*/*B* ratio and ALSFRS-R score for bulbar-onset ALS patients alone. In bulbar-onset ALS patients, lower oral *F*/*B* ratio was strongly associated with lower ALSFRS-R score (greater ALS severity). **G** Linear regression analysis comparing oral *F*/*B* ratio and bulbar/respiratory ALSFRS-R subscore for spinal-onset ALS patients alone. Bulbar/respiratory ALSFRS-R subscore (maximum score of 24) measures the severity of bulbar/respiratory symptoms. Lower subscore indicates more severe bulbar/respiratory symptoms[7]. Spinal-onset ALS patients displayed no significant association between oral *F*/*B* ratio and bulbar/respiratory ALSFRS-R subscore. **H** Linear regression analysis comparing oral *F*/*B* ratio and bulbar/respiratory ALSFRS-R subscore for bulbar-onset ALS patients alone. In bulbar-onset ALS patients, lower oral *F*/*B* ratio was strongly associated with lower bulbar/respiratory ALSFRS-R subscore. Statistics: Statistical significance was established using linear regression analysis and Spearman’s correlation coefficient (*r*) test

We next investigated if the degree of dysbiosis can predict ALS severity. Overall, ALS patients showed a significant linear trend between gut-dysbiosis (a shift to higher fecal *F/B-*ratio) and disease severity (ALSFRS-R score) (*r* = 0.5722. *P* = 0.0003; Fig. 5A). However, we observed highly distinct patterns between sALS patients and bALS patients (Fig. 5A). While sALS patients showed a strong correlation between gut-dysbiosis and ALS severity (*r* = 0.9470, *P* < 0.0001; Fig. 5B), bALS patients did not (*r* = 0.0052, *P* = 0.9881; Fig. 5C). sALS patients with ALSFRS-R scores of 40–48 (minimal symptoms) displayed a comparable fecal *F/B-*ratio to healthy control ≈0.5. sALS patients with ALSFRS-R scores of 30–40 (moderate symptoms) consistently displayed a fecal *F/B-*ratio of 1–2. sALS patients with ALSFRS-R scores < 30 (severe symptoms) consistently showed a fecal *F/B-*ratio of 2–4 (Fig. 5B). For bALS patients, fecal *F/B-*ratio remained at ≈0.5 for all ALSFRS-R scores (Fig. 5C). Unlike gut-dysbiosis, oral-dysbiosis (a shift to lower oral *F/B-*ratio) was not significantly correlated with disease severity for ALS patients overall (*r* = 0.2046, *P* = 0.2313; Fig. 5D). However, surprisingly, when the data were divided based on ALS subtypes, lower oral *F/B-*ratio (greater oral-dysbiosis) was strongly associated with greater disease severity in bALS patients (*r* = 0.9842, *P* < 0.0001; Fig. 5F) but not in sALS patients (*r* = 0.0481, *P* = 0.8236; Fig. 5E). Together, our data suggest that gut-dysbiosis (a shift in fecal *F/B-*ratio) predicts disease severity of sALS, whereas oral-dysbiosis (a shift in oral *F/B-*ratio) predicts disease severity of bALS.

There are many confounders which can influence microbiome studies, such as clinical risk factors and genetic background. This issue is particularly prevalent in ALS microbiome studies as ALS is a rare and rapidly progressive disease (5.2 cases per 100,000; mean survival from onset: 20–48 months) [58]. Our study encompasses 36 ALS patients (24 sALS patients and 12 bALS patients) and 20 controls. This is consistent in size with other ALS microbiome studies: 37 ALS patients and 29 controls were examined in [33], 6 ALS patients and 5 controls were examined in [36], and 25 ALS patients and 32 controls were examined in [37]. The largest ALS microbiome study to date examined just 50 ALS patients and 50 controls [59]. We have attempted to minimize confounding effects from a relatively small sample size through rigorous exclusion criteria and the use of spouses as controls to account for environmental factors, but we cannot completely discount this possibility. We perform a number of analyses below to attempt to account for many potential confounding effects.

### Evaluation of potential confounding factors

A concern for this study is that oral-microbiome changes we found in bALS might represent a symptom of the disease rather than a pathological driver. Patients with bulbar symptoms experience difficulty swallowing and pooling of saliva [60], which may cause symptom-induced changes in oral-microbiome. While this is a potential confounder, both sALS and bALS patients in our study displayed bulbar symptoms due to the progression of disease and the spread of paralysis (*P* = 0.5361; Fig. 1B). Additionally, despite clear bulbar/respiratory symptoms in both groups, we observed a strong association between oral *F/B-*ratio and bulbar/respiratory symptom severity in only bALS patients (*r* = 0.8873, *P* = 0.0003; Fig. 5H) with no significant association in sALS patients (*r* = 0.1200, *P* = 0.5766; Fig. 5G). Several sALS patients in our study presented comparably severe bulbar/respiratory symptoms as bALS patients, but these patients still did not show oral-dysbiosis and maintained oral *F/B-*ratio of ≈1 as in healthy controls (Fig. 5G, H). Thus, our finding of oral-dysbiosis in bALS patients but not sALS patients is unlikely a symptom artifact and suggests that oral-dysbiosis may be a pathological driver of bALS.

We also considered the possibility that the oral dysbiosis we observed in bALS patients may be driven by differences in oral health between our groups. To investigate this, we examined a number of markers of caries and periodontal disease. We first examined salivary pH; low pH (fast rates of acid production) has previously been linked with the presence of caries and periodontal disease [61]. We did not see significant differences in salivary pH in our patients (Supplemental Fig. 9A). We next examined MUC7 levels. MUC7 is a predominant mucin in saliva. Low levels of MUC7 are found to be associated with elevated *Streptococcus mutans* titers [62]. For this reason, MUC7 is thought to potentially serve as a predictor of caries risk assessment for older adults [62]. We saw no significant differences in levels of MUC7 in our patient pools (Supplemental Fig. 9B). Finally, we examined levels of *Streptococcus mutans*, *Lactobacillus,* and *Candida albicans* with qPCR. A number of studies have reported increases in these to be correlated with increases in caries initiation and progression [63–66]. We did not observe any significant differences in proportional levels of *S. mutans*, *Lactobacillus,* or *C. albicans* (Supplemental Fig. 9C, D, E). Based on these findings, we do not observe evidence for differences in the prevalence of caries or periodontal diseases between controls, sALS or bALS patients that could confound our conclusions.

We also considered the possibility that the differences we observed might be impacted by confounding effects from gender, age, BMI, or disease duration. To evaluate potential effects from gender, we repeated our key analyses, evaluating males and females separately. We observed our key conclusions to be recapitulated both in the males only and female only data sets (Supplemental Figs. 10, 11). To evaluate potential confounding effects from age, BMI, or disease duration, we repeated our regression analyses examining covariance between ALSFRS-R in sALS patients adjusted for age, BMI, or disease duration against fecal F/B ratio adjusted for age, BMI, or disease duration (Supplemental Figs. 12). We also evaluated covariance between ALSFRS-R in bALS patients adjusted for age, BMI, or disease duration against oral F/B ratio adjusted for age, BMI, or disease duration (Supplemental Fig. 13). In all cases, our conclusions were maintained after corrections for these covariates.

### For spinal-onset ALS, gut-dysbiosis may drive microbial translocation to blood, leading to increased disease severity, whereas for bulbar-onset ALS, oral-dysbiosis may drive microbial translocation to blood, leading to increased disease severity

**Fig. 6:**
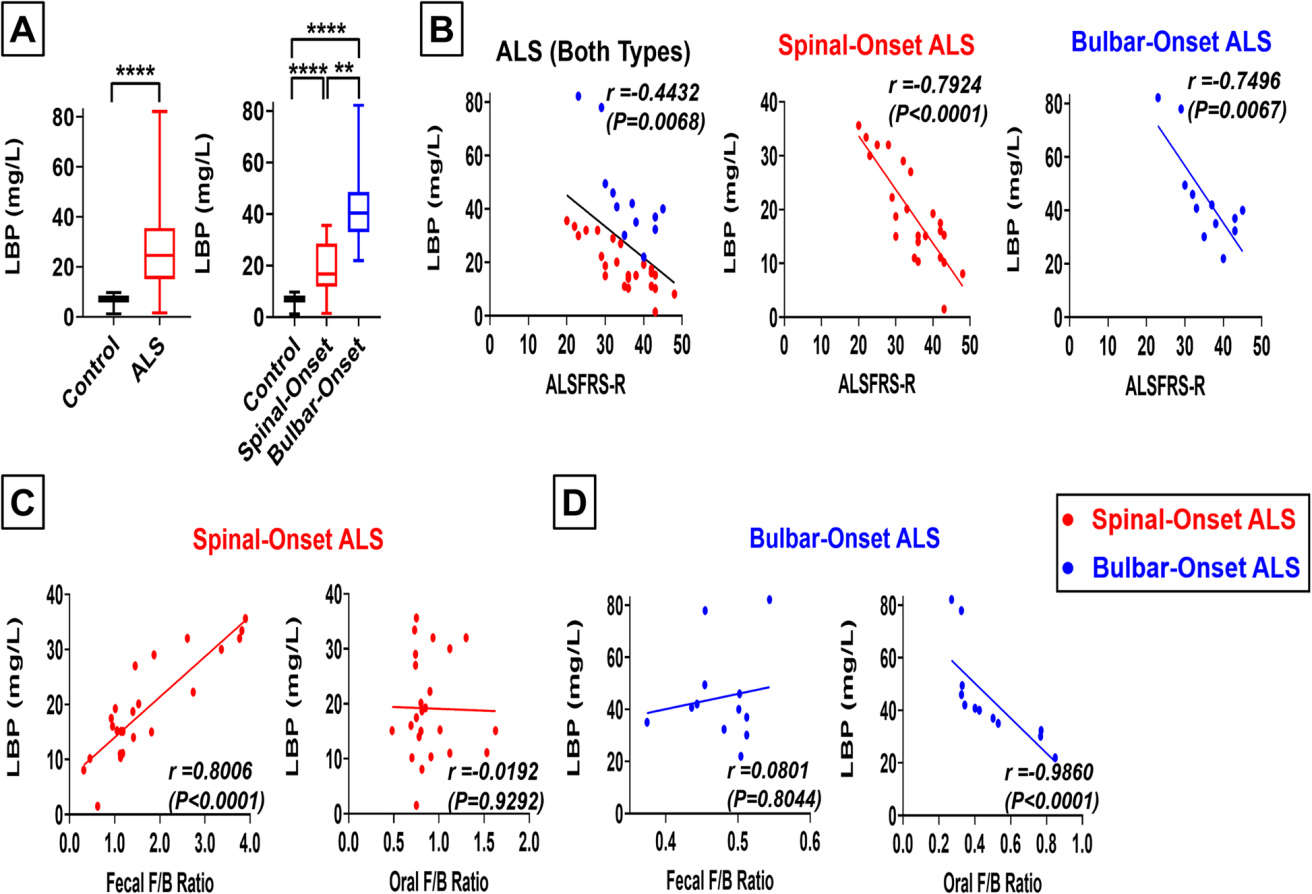
For Spinal-Onset ALS, Gut-Dysbiosis May Drive Microbial Translocation to Blood, Leading to Increased Disease Severity, whereas for Bulbar-Onset ALS, Oral-Dysbiosis May Drive Microbial Translocation to Blood, Leading to Increased Disease Severity. **A** Blood lipopolysaccharide-binding protein (LBP) levels in mg/L as a marker for microbial translocation to the blood. Results represent both ALS patients (spinal- and bulbar-onset ALS patients combined) or spinal- and bulbar-onset ALS patients quantified separately. Both spinal- and bulbar-onset ALS patients displayed significant increases in blood LBP levels, with bulbar-onset ALS patients showing significantly higher levels than spinal-onset ALS patients. **B** Linear regression analysis comparing blood LBP levels and ALSFRS-R score for all ALS patients combined (left), spinal-onset ALS patients only (middle), and bulbar-onset ALS patients only (right). Spinal-onset ALS patients shown in red, bulbar-onset ALS patients shown in blue. In both spinal- and bulbar-onset ALS patients, higher blood LBP levels (greater microbial translocation to the blood) were strongly associated with lower ALSFRS-R score (greater ALS severity). **C** Linear regression analyses comparing fecal *F*/*B* ratio and blood LBP levels (left) and oral *F*/*B* ratio and blood LBP levels (right) in spinal-onset ALS patients. In spinal-onset ALS patients, fecal *F*/*B* ratio showed strong correlations with blood LBP levels, but oral *F*/*B* ratio showed poor correlations with blood LBP levels. In spinal-onset ALS patients, higher fecal *F*/*B* ratio (greater gut-dysbiosis) was strongly associated with greater blood LBP levels (greater microbial translocation to the blood). **D** Linear regression analyses comparing fecal *F*/*B* ratio and blood LBP levels (left) and oral *F*/*B* ratio and blood LBP levels (right) in bulbar-onset ALS patients. In bulbar-onset ALS patients, oral *F*/*B* ratio showed strong correlations with blood LBP levels, but fecal *F*/*B* ratio showed poor correlations with blood LBP levels. In bulbar-onset ALS patients, lower oral *F*/*B* ratio (greater oral-dysbiosis) was strongly associated with greater blood LBP levels (greater microbial translocation to the blood). Statistics: In panel A, contrasts between control and ALS patients (combined) utilized Mann–Whitney test, while contrasts between control, spinal-onset ALS, and bulbar-onset ALS utilized Kruskal–Wallis test with Dunn’s post-hoc test. Values represent sample median with interquartile range. For panels B-D, statistical significance was established using linear regression analysis and Spearman’s correlation coefficient (*r*) test. *P < 0.05, **P < 0.01, ***P < 0.001, ****P < 0.0001

Microbial-dysbiosis can weaken organ barriers, causing microbes to translocate to the blood and affect nearby tissues [11, 67]. We hypothesized that oral-dysbiosis may cause microbial translocation to blood vessels/nerves supplying head and neck muscles first, driving bALS pathologies. On the other hand, gut-dysbiosis may cause microbial translocation to blood vessels/nerves supplying limb muscles first, driving sALS pathologies. To test this possibility, we measured blood lipopolysaccharide-binding protein (LBP) levels, a marker for microbial translocation to the blood [68]. To more directly evaluate blood microbial translocation, we also measured the abundance of microbial species in the blood through qPCR of plasma 16S rDNA [69]. Plasma LBP and 16S rDNA levels were significantly increased in both sALS and bALS patients (Fig. 6A, Supplemental Fig. 14A), indicating greater microbial translocation to blood. We show increases in these markers of microbial translocation to blood to be correlated with more severe symptoms in both sALS patients (*r* = 0.7924, *P* < 0.0001) and bALS patients (*r* = 0.7496, *P* = 0.0067; Fig. 6B, Supplemental Fig. 14B). Interestingly, in sALS patients, gut-dysbiosis (a shift in fecal *F/B-*ratio) was strongly associated with increases in markers of microbial translocation to blood (*r* = 0.8006, *P* < 0.0001), but oral-dysbiosis (a shift in oral *F/B-*ratio) was not (*r* = 0.0192, *P* = 0.9292; Fig. 6C-D, Supplemental Fig. 14C). However, in bALS patients, oral-dysbiosis was strongly associated with greater microbial translocation to blood (*r* = 0.9860, *P* < 0.0001), whereas gut-dysbiosis was not (*r* = 0.1926, *P* = 0.5462; Fig. 6C-D, Supplemental Fig. 14D). These results suggest that gut-dysbiosis may facilitate local microbial translocation to cause pathologies in sALS patients, while oral-dysbiosis may facilitate local microbial translocation to cause pathologies in bALS patients. These trends were preserved in matched-pair analyses comparing patients to matched household controls (Supplemental Fig. 3I). Additionally, our conclusions were maintained after corrections for age, BMI, or disease duration (Supplemental Figs. 15–16).

Although we demonstrated increased blood microbial translocation in ALS patients through measures of plasma LBP and qPCR confirmation of increased 16S rDNA, we were unable to provide an accurate report on the taxons which are translocating to blood because the 16S/18S rDNA ratio in blood was dramatically lower than that in saliva and stool samples. For this reason, the accuracy of next generation sequencing is expected to be too low to provide reliable data.

### High fecal *Fusobacteria* abundance is positively correlated with microbial translocation in spinal-onset ALS, whereas high oral *Fusobacteria* abundance is positively correlated with microbial translocation in bulbar-onset ALS

**Fig. 7:**
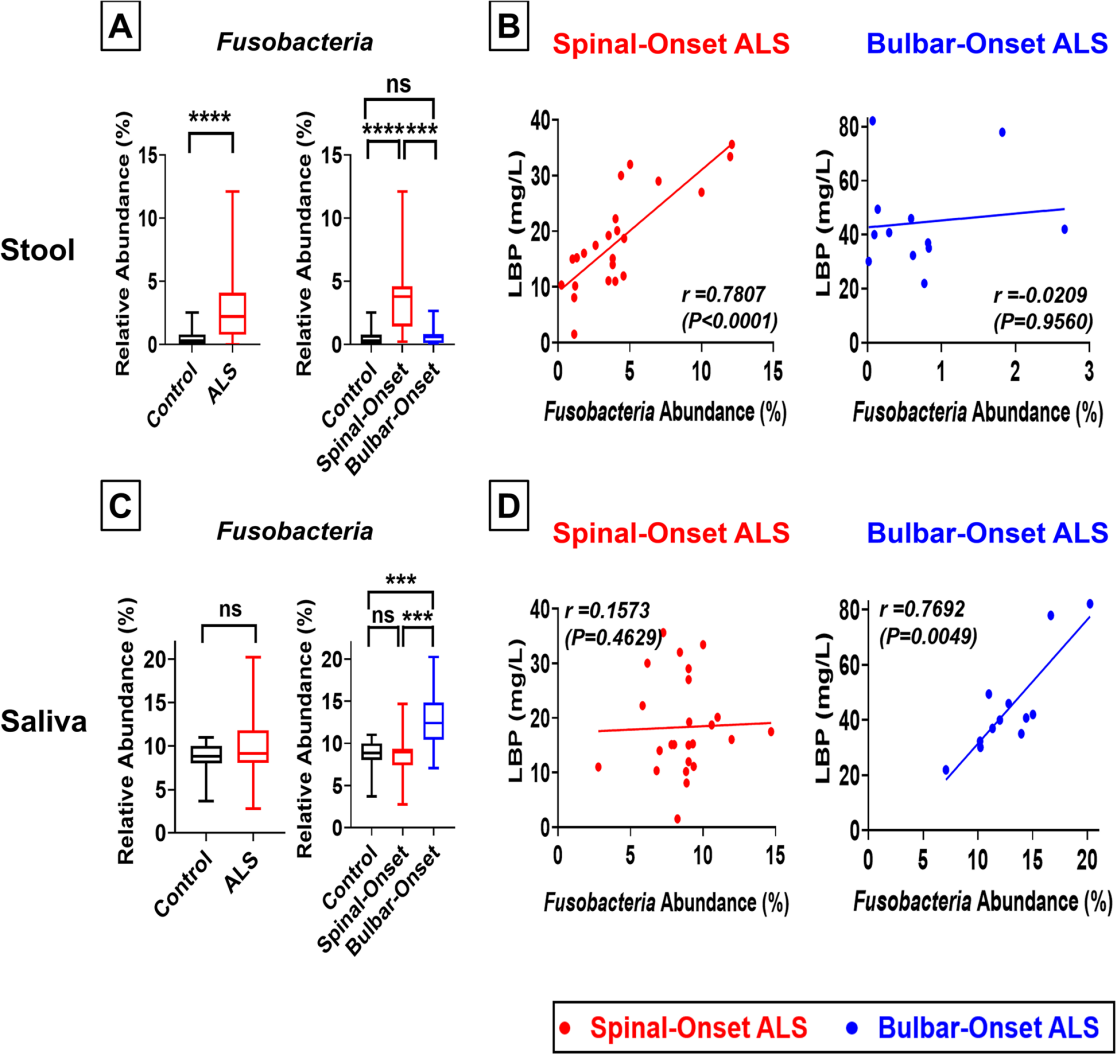
High Fecal *Fusobacteria* Abundance is Positively Correlated with Microbial Translocation in Spinal-Onset ALS, whereas High Oral *Fusobacteria* Abundance is Positively Correlated with Microbial Translocation in Bulbar-Onset ALS. **A** Percent abundance of *Fusobacteria* in gut microbiome. Results represent both ALS (spinal- and bulbar-onset ALS combined) or spinal- and bulbar-onset ALS quantified separately. Spinal-onset ALS patients displayed a significant enrichment of *Fusobacteria* in the gut. No significant change in bulbar-onset ALS. **B** Linear regression analysis comparing fecal *Fusobacteria* abundance and blood LBP levels in spinal-onset ALS patients (left) and bulbar-onset ALS patients (right). We observed a strong correlation between fecal *Fusobacteria* abundance and LBP levels in spinal-onset ALS patients but not in bulbar-onset ALS patients. **C** Percent abundance of *Fusobacteria* in oral microbiome. Bulbar-onset ALS patients displayed a significant enrichment of *Fusobacteria* in saliva. No significant change in spinal-onset ALS. **D** Linear regression analysis comparing oral *Fusobacteria* abundance and blood LBP levels in spinal-onset ALS patients (left) and bulbar-onset ALS patients (right). We observed a strong correlation between oral *Fusobacteria* abundance and LBP levels in bulbar-onset ALS patients but not in spinal-onset ALS patients. Statistics: In panel A and C, contrasts between control and ALS patients (combined) utilized Mann–Whitney test, while contrasts between control, spinal-onset ALS, and bulbar-onset ALS utilized Kruskal–Wallis test with Dunn’s post-hoc multiple comparisons test. Values represent sample median with interquartile range. For panels B and D, statistical significance was established using linear regression analysis and Spearman’s correlation coefficient (*r*) test. *P < 0.05, **P < 0.01, ***P < 0.001, ****P < 0.0001

*Fusobacteria* is described as an “enabler” for other microbes to translocate to the blood because it can disrupt endothelial cell junctions and increase blood vessel permeability to microbes [70]. Consistent with strong associations between gut-dysbiosis and microbial translocation in sALS (Fig. 6), fecal *Fusobacteria* abundance was significantly increased in sALS but not in bALS (Fig. 7A, Supplemental Fig. 3, 4). Further, fecal *Fusobacteria* abundance was strongly associated with microbial translocation in sALS (*r* = 0.7807, *P* < 0.0001; Fig. 7B) but not in bALS (*r* = 0.0209, *P* = 0.9560; Fig. 7B). In contrast, oral *Fusobacteria* abundance was significantly increased in bALS but not in sALS (Fig. 7C, Supplemental Fig. 3, 4). Additionally, oral *Fusobacteria* abundance was strongly associated with microbial translocation in bALS (*r* = 0.7692, *P* = 0.0049; Fig. 7D) but not in sALS (*r* = 0.1573, *P* = 0.4629; Fig. 7D). These data are consistent with strong associations between oral-dysbiosis and microbial translocation in bALS (Fig. 6). Our findings suggest that a fecal enrichment of *Fusobacteria* may facilitate gut-dysbiosis-induced microbial translocation in sALS, whereas an oral enrichment of *Fusobacteria* may facilitate oral-dysbiosis-induced microbial translocation in bALS. These trends were preserved in matched-pair analyses comparing patients to household controls (Supplemental Fig. 3G, H). Additionally, our conclusions were maintained after adjusting for age, BMI, or disease duration (Supplemental Figs. 17–18). These results were also independently validated by qPCR (Supplemental Fig. 4).

## Discussion

Our results show clear microbiome differences between sALS and bALS patients. We saw significant gut-microbiome changes (gut-dysbiosis) in sALS patients but not in bALS patients. Conversely, we observed significant oral-microbiome changes (oral-dysbiosis) in bALS patients but not in sALS patients. When we pooled all ALS patients, we found gut-microbiome changes to be driven entirely by the abundance of sALS patients in the group while oral-microbiome differences to be driven entirely by the abundance of bALS patients in the group. Most microbiome studies have not stratified ALS patients by subtypes [33, 36, 37] and thus will have a variable makeup of the proportion of sALS compared to bALS. This may explain the inconsistencies of prior ALS studies, where gut-microbiome changes have been reported in some studies [33, 36] but not others [37].

We saw a strong correlation between microbial-dysbiosis and ALS severity with distinct differences between sALS and bALS. We found increasing gut-dysbiosis with worsening symptoms in sALS and increasing oral-dysbiosis with worsening symptoms in bALS. In both cases, the associations were very strong (sALS: *r* = 0.9470, *P* < 0.0001; bALS: *r* = 0.9842, *P* < 0.0001). However, oral-dysbiosis failed to predict disease severity of sALS, and gut-dysbiosis failed to predict disease severity of bALS. Additionally, oral-dysbiosis in bALS is unlikely a symptom artifact because sALS patients with comparably severe oral/bulbar/respiratory symptoms as bALS patients showed no oral-dysbiosis. These results suggest different microbial pathological processes to be driving two ALS subtypes.

Our study demonstrates the shift in fecal *Firmicutes* to *Bacteroidetes* (*F*/*B*) to be a major driver of gut-dysbiosis in sALS patients and the shift in oral *F*/*B* to be a main driver of oral-dysbiosis in bALS patients. In both cases, we show the magnitude of this shift to be strongly correlated with increased microbial translocation and greater disease severity. This is consistent with two prior small studies reporting altered *F*/*B* ratio in ALS patients. [36, 71]. Consistent with the findings of our study, a recent study also showed that higher fecal *F*/*B* ratio was associated with increased risk of death in ALS patients [72]. Additionally, we saw enrichment of *Fusobacteria* in stool and saliva samples of sALS and bALS patients, respectively. *Fusobacteria* is known to increase local endothelial permeability, facilitate other microbes to translocate to nearby tissues, and cause pathologies [70]. Thus, fecal/oral enrichment of *Fusobacteria* may exacerbate local microbial translocation triggered by the shift in fecal/oral *F*/*B* and contribute to sALS and bALS pathologies, respectively.

**Fig. 8:**
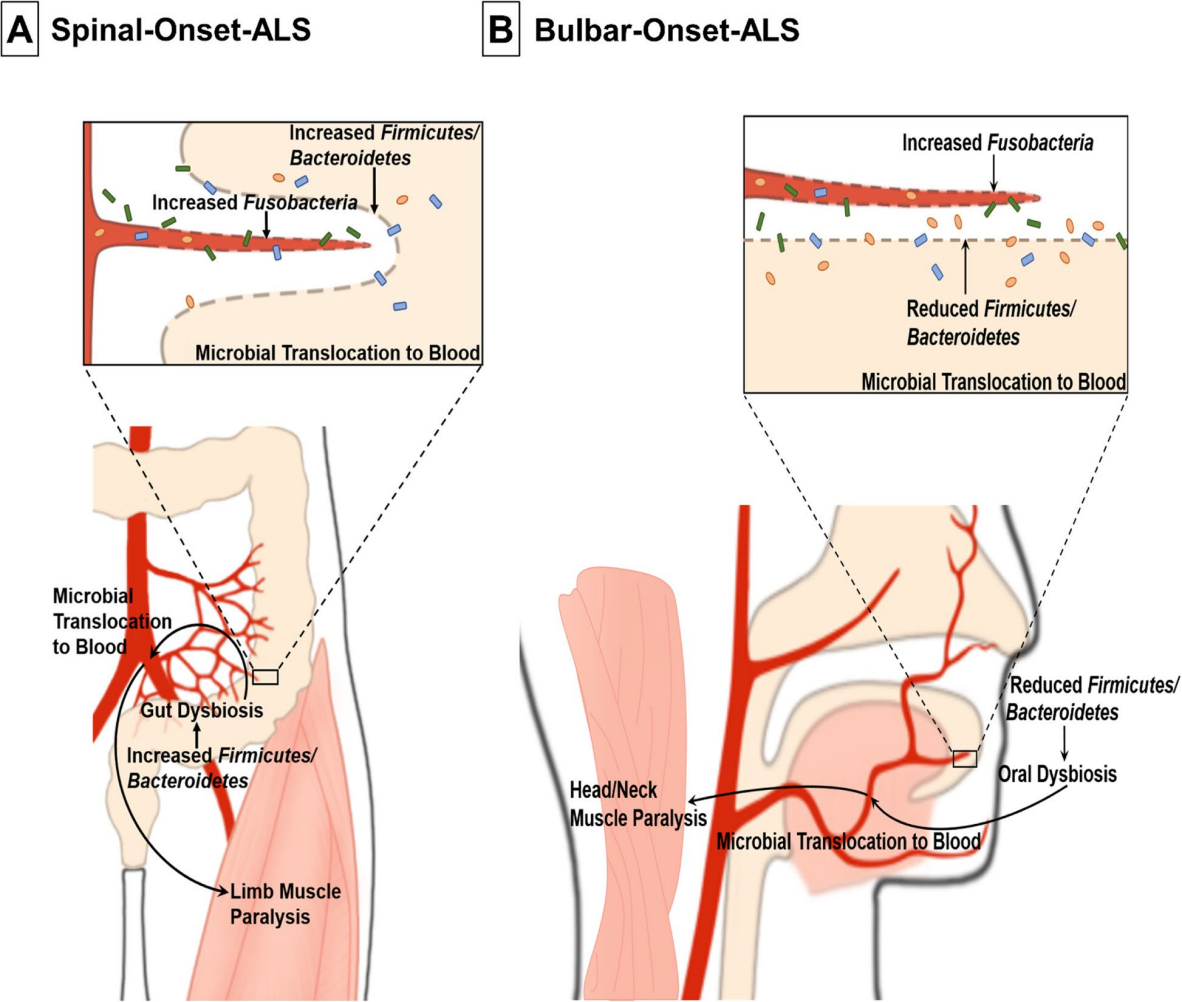
Schematic Diagram of Proposed Mechanisms for Spinal- and Bulbar-Onset ALS Pathogenesis. **A** Proposed mechanism for the pathogenesis of spinal-onset ALS. A shift to higher gut *Firmicutes*/*Bacteroidetes* (*F*/*B*) ratio causes gut-dysbiosis. Gut-dysbiosis then weakens gut-barriers via local inflammation, enabling microbes to cross the mucosal barriers, translocate to local blood vessels/nerves supplying limb muscles first, and cause limb paralysis. Once high levels of *Fusobacteria* cross weakened gut-barriers thanks to gut-dysbiosis, they increase endothelial permeability to microbes, exacerbate local microbial translocation to the blood, and worsen spinal-onset ALS pathologies. **B** Proposed mechanism for the pathogenesis of bulbar-onset ALS. A shift to lower oral *F*/*B* ratio causes oral-dysbiosis. Oral-dysbiosis then weakens oral cavity barriers via local inflammation, enabling microbes to cross the mucosal barriers, translocate to local blood vessels/nerves supplying head and neck muscles first, and trigger their paralysis. Once high levels of *Fusobacteria* cross weakened oral cavity barriers thanks to oral-dysbiosis, they increase endothelial permeability to microbes, exacerbate local microbial translocation to the blood, and worsen bulbar-onset ALS pathologies

Gut-dysbiosis and oral-dysbiosis may represent a pathological driver of sALS and bALS, respectively. High fecal *F/B-*ratio and gut-dysbiosis have been shown to decrease limb muscle fiber size and cause limb muscles to become insulin-resistant and favor lipid metabolism [73–75], all of which are evident features of sALS [76, 77]. Additionally, patients manifesting gut-dysbiosis often experience limb weakness due to pathologies in blood vessels supplying limb muscles, potentially triggered by gut dysbiosis-induced microbial translocation to the vasculature [78–82]. Thus, our findings of gut-dysbiosis in sALS and its strong association with microbial translocation to blood and disease severity suggest the following as the potential mechanism for sALS pathogenesis: a shift to higher fecal *F/B-*ratio causes gut-dysbiosis, enabling microbes to cross gut-barriers, translocate to blood vessels/nerves supplying limb muscles first, and trigger limb paralysis (Fig. 8A). Additionally, once high levels of gut *Fusobacteria* cross weakened gut-barriers thanks to gut-dysbiosis, they can increase endothelial permeability to microbes, exacerbate local microbial translocation to the blood, and worsen sALS pathologies (Fig. 8A). This hypothesis is further supported by a somewhat direct/close anatomic connection between gut and lower limbs. Common iliac arteries, which provide the primary blood supply to the lower limbs, are overlaid by the small intestine and covered by the peritoneum in front and medially. It is thus possible that leaked gut microbes may translocate to vessels/nerves supplying lower limbs and initiate pathologies. The connection is little more distant for upper limbs, but several lines of evidence suggest that pathologies from the gut may lead to pathologies in upper limbs as well. For example, patients with inflammatory bowel disease often exhibit lower and upper limb numbness/weakness/pain due to pathologies in distal branches of iliac arteries supplying lower limbs and subclavian arteries supplying upper limbs [78, 79, 81].

While in bALS, oral-dysbiosis driven by decreased oral *F*/*B*-ratio may enable microbial translocation to blood vessels/nerves supplying head/neck muscles first, leading to their paralysis (Fig. 8B). Once high levels of oral *Fusobacteria* cross weakened oral cavity barriers thanks to oral-dysbiosis, they can exacerbate local microbial translocation and worsen bALS pathologies (Fig. 8B). In support of this, for bALS patients, we found that oral-dysbiosis, but not gut-dysbiosis, is associated with greater microbial translocation to blood and more severe symptoms. Additionally, oral-dysbiosis-induced microbial translocation (which we observed in bALS patients) is reported to cause head/neck pathologies [83]. Moreover, the major oral-microbiome altered in bALS were *Veillonellaceae* and *Prevotellaceae* (Fig. 4B), which regulate contractile functions of nearby skeletal muscles by controlling salivary nitrate/nitric oxide synthesis [84, 85].

ALS is a motor neuron disease that impacts not only skeletal muscles but also lower and upper motor neurons. Several studies suggest that skeletal muscles can be pathologically affected in ALS first, which then drive degeneration of lower and upper motor neurons that innervate the affected muscles [86, 87]. For example, in ALS mice, skeletal muscles showed pathological changes long before lower and upper motor neurons were pathologically affected and neurodegeneration took place [87]. Additionally, skeletal muscle-specific overexpression of mutant superoxide mutase-1 (SOD1), the gene associated with familial ALS, was sufficient to cause not only muscle weakness but also loss of lower/upper motor neurons, brain pathologies, and axonopathy [86]. Thus, gut- and oral-dysbiosis, although impacting peripheral skeletal muscles first, may also drive pathologies in brain/nervous system innervating these muscles to cause sALS and bALS.

Gut- and oral-dysbiosis may first impact limbs and head/neck muscles via local microbial translocation, respectively, but translocated microbes can spread systemically in the end to cause pathologies in other body regions. Indeed, regardless of where the muscle weakness first appears in ALS, muscle atrophy/paralysis spreads to other parts of the body as the disease progresses [1, 5]. In other words, sALS patients, although presenting symptoms in limb muscles initially, may eventually develop symptoms in head and neck muscles. Similarly, bALS patients, although presenting symptoms first in head/neck muscles, may eventually experience weakness in limb muscles. Longitudinal microbiome studies with sALS and bALS patients to uncover mechanisms how translocated microbes spread to other body regions and how subsequent muscle pathologies spread to various brain regions may help us identify therapeutic targets to slow down the spread of paralysis.

Our study suggests correcting gut-dysbiosis as a therapeutic strategy for sALS patients and correcting oral-dysbiosis as a therapeutic strategy for bALS patients. In sALS patients, higher fecal *F/B-*ratio was able to predict greater microbial translocation to blood and greater disease severity. In contrast, in bALS patients, lower oral *F/B-*ratio was able to predict greater microbial translocation to blood and greater disease severity. Thus, targeting *F/B-*ratio in opposite directions in different organs (gut vs. oral cavity) based on locations of disease onset might be a viable therapeutic strategy for sALS and bALS. Several therapeutic interventions have been developed to alter microbiota composition in the gut and the oral cavity. For example, fecal transplantation, antibiotics, and probiotics were able to correct gut-dysbiosis and alleviate pathologies in AD and PD animal models [16, 19–21]. On the other hand, probiotics that rapidly dissolve in the oral cavity were able to successfully correct oral-dysbiosis without affecting gut-microbiome [88, 89]. Examining the effects of these interventions on disease severity in sALS and bALS patients may enable us to develop therapy targeting specific ALS subtypes.

## Conclusions

This study shows clear microbiome differences between ALS subtypes with gut-microbiome changes in sALS and oral-microbiome changes in bALS. In sALS, gut-dysbiosis, but not oral-dysbiosis, was strongly associated with microbial translocation to blood and more severe symptom presentations. In contrast, in bALS, oral-dysbiosis, but not gut-dysbiosis, was strongly associated with microbial translocation to blood and greater disease severity. In both sALS and bALS, greater microbial translocation was associated with more severe symptoms. Gut- and oral-dysbiosis-induced microbial translocation may damage different local tissues and underlie clinico-pathological differences observed between the two ALS subtypes. Our findings support that these ALS subtypes should be considered distinct diseases and treated separately. Our study raises microbiome manipulation as a potential therapy targeting specific ALS subtypes.

## Supplementary Information

**Additional file 1.**

## Data Availability

Raw, de-identified data are available from the corresponding author on reasonable request.

## Abbreviations

16S rRNA: 16S ribosomal Ribonucleic Acid
16S rDNA: 16S ribosomal Deoxyribonucleic Acid
ALS: Amyotrophic Lateral Sclerosis
ALSFRS-R: Amyotrophic Lateral Sclerosis Functional Rating Scale-Revised
bALS: Bulbar-onset Amyotrophic Lateral Sclerosis
sALS: Spinal-onset Amyotrophic Lateral Sclerosis
*F*/*B* ratio: *Firmicutes* To *Bacteroidetes* ratio
FDA: Food and Drug Association
LBP: Lipopolysaccharide-Binding Protein
PCA: Principle Component Analysis
PCR: Polymerase Chain Reaction
qPCR: Quantitative Polymerase Chain Reaction
OTU: Operational Taxonomic Units
SOD1: Superoxide Dismutase 1
