# Supplementary material for: Gut- and oral-dysbiosis differentially impact spinal- and bulbar-onset ALS, predicting ALS severity and potentially determining the location of disease onset
Source: BMC Neurol. 2022 Feb 21;22:62. doi: 10.1186/s12883-022-02586-5 (PMC8862222; doi:10.1186/s12883-022-02586-5)
Supplement: Supplementary file 1 — Additional file 1. [file 12883_2022_2586_MOESM1_ESM.pdf]

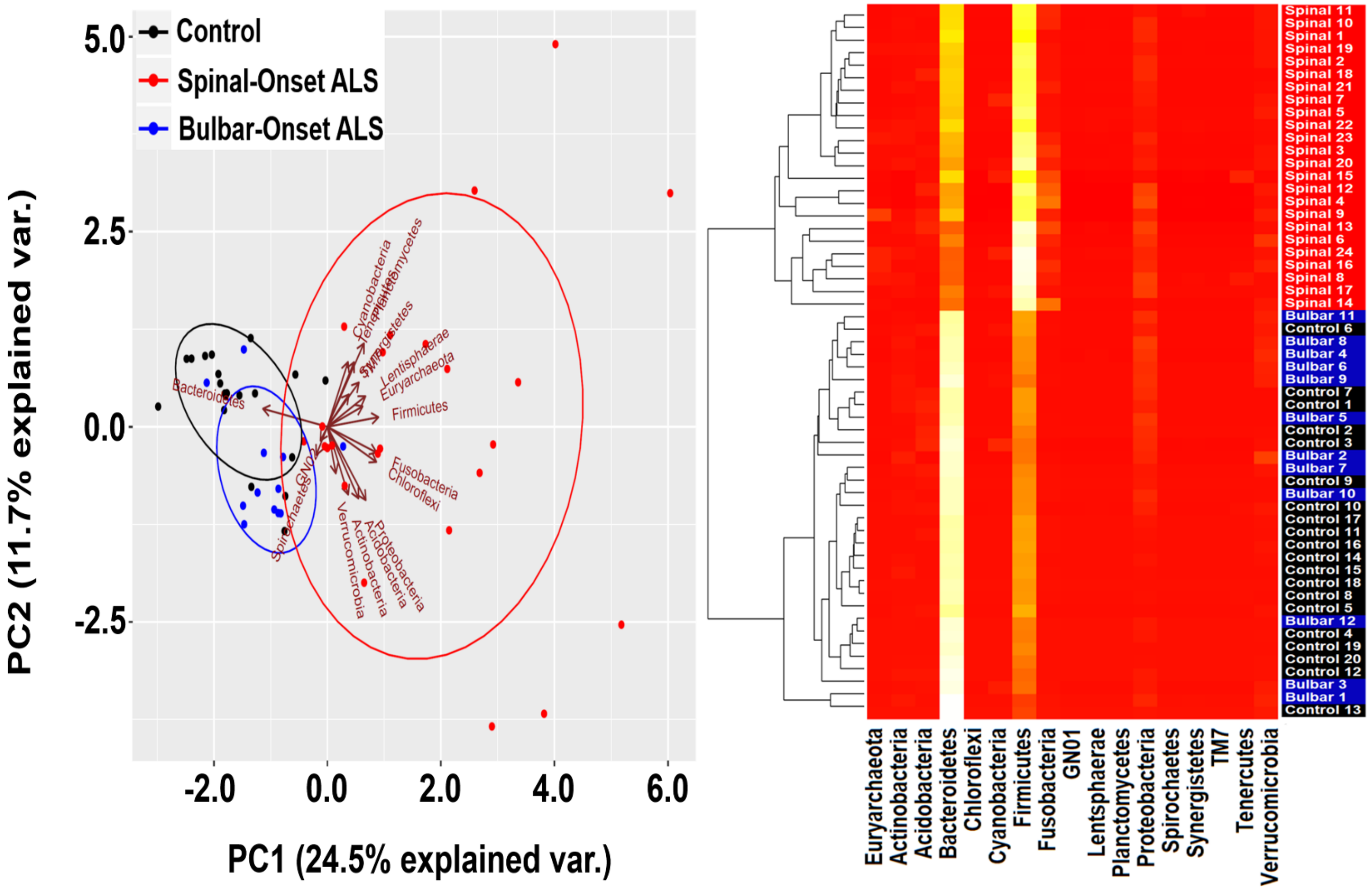

Supplemental Figure 1. In Gut Microbiome, Spinal-Onset ALS Patients Formed a Distinct Cluster from Controls and Bulbar-Onset ALS patients. Left panel represents the principal component analysis of gut-microbiome species taxa at the phylum level. Data derived from 20 healthy controls (black), 24 spinal-onset ALS patients (red), and 12 bulbar-onset ALS patients (blue). The phyla variables separating the groups are indicated as axes. Right panel represents the heatmap for gut-microbiome distribution at the phylum level. **Statistics:** Clustering was performed based on Bray Similarity matrix.

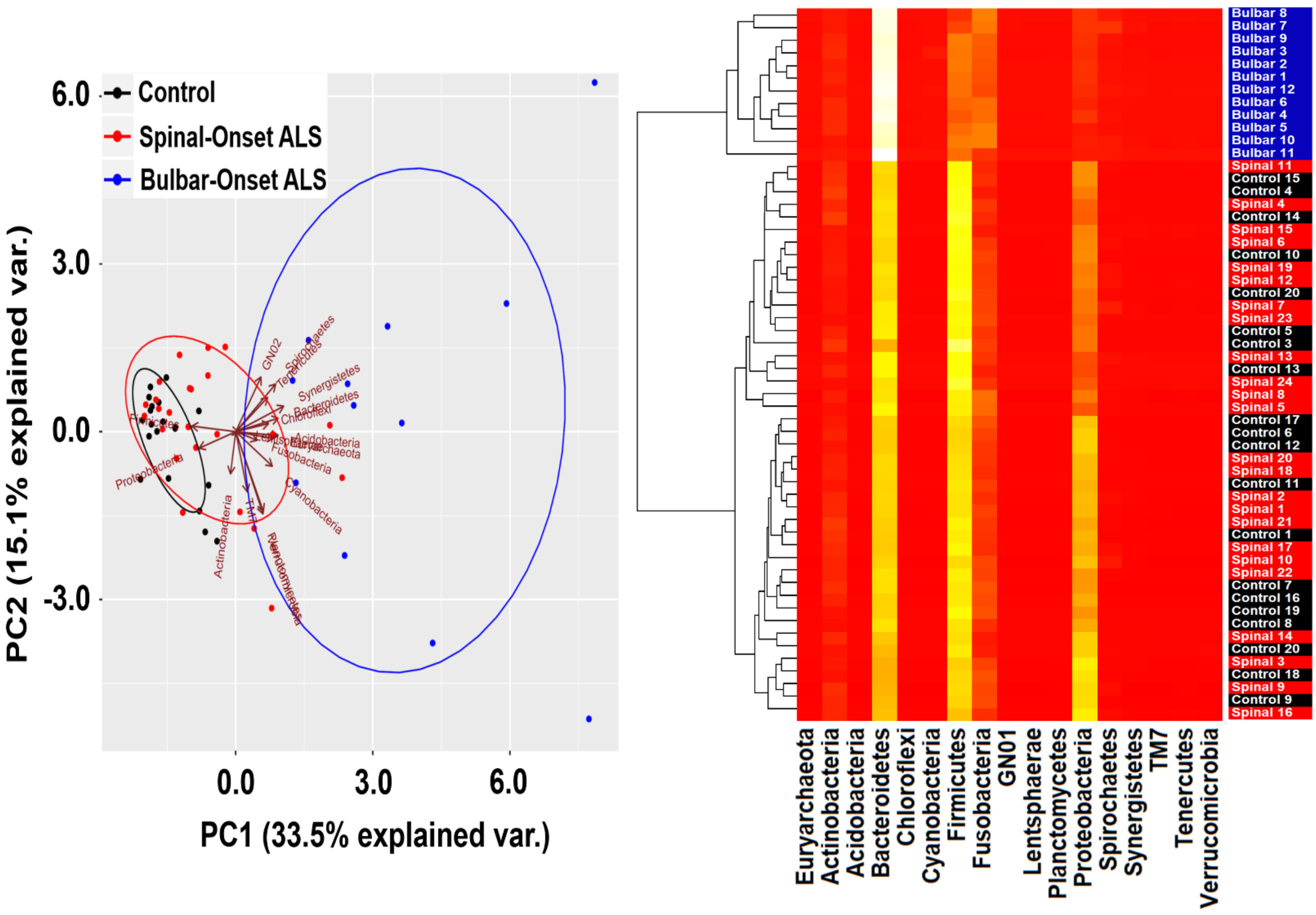

Supplemental Figure 2. In Oral Microbiome, Bulbar-Onset ALS Patients Formed a Distinct Cluster from Controls and Spinal-Onset ALS patients. Left panel represents the principal component analysis of oral-microbiome species taxa at the phylum level. Data derived from 20 healthy controls (black), 24 spinal-onset ALS patients (red), and 12 bulbar-onset ALS patients (blue). The phyla variables separating the groups are indicated as axes. Right panel represents the heatmap for oral-microbiome distribution at the phylum level. **Statistics:** Clustering was performed based on Bray Similarity matrix.

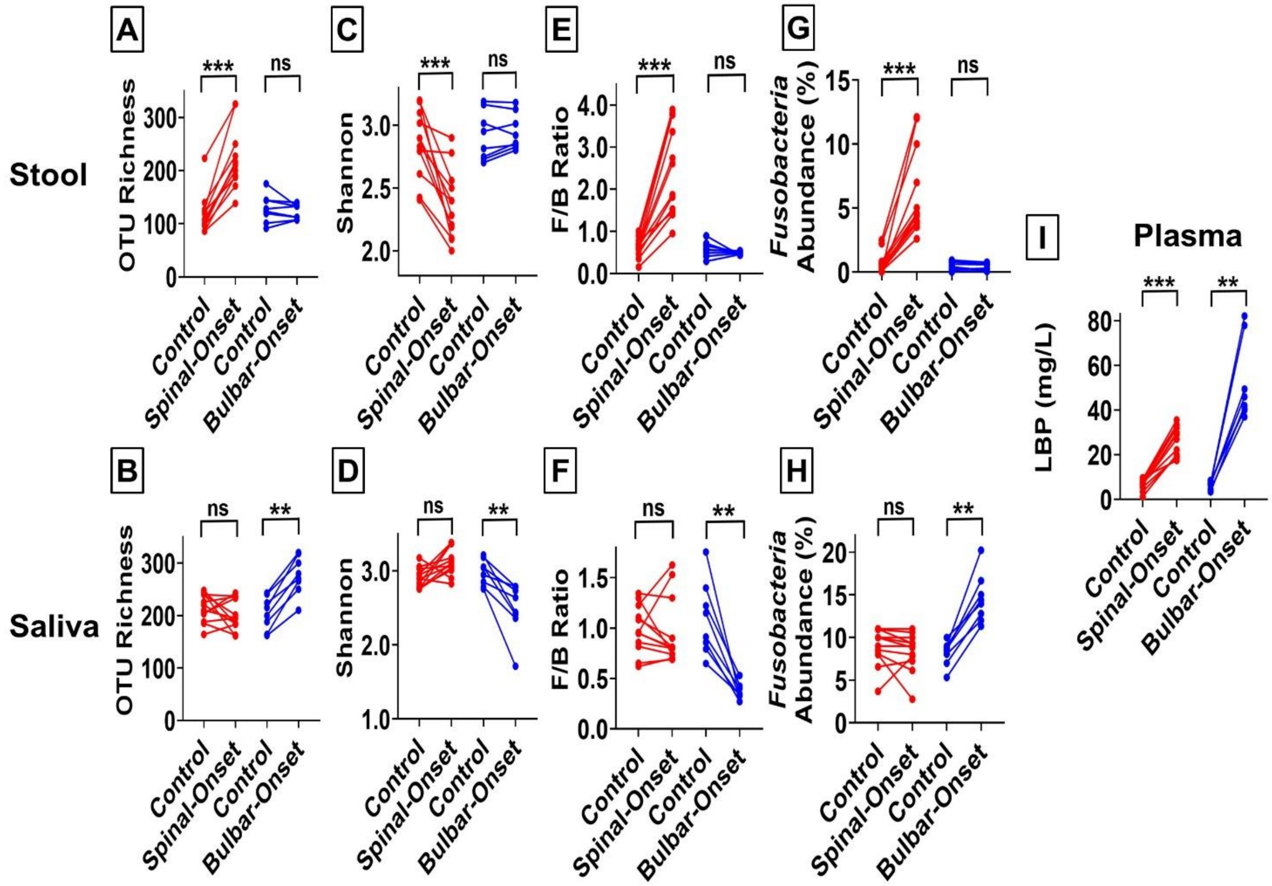

Supplemental Figure 3. OTU Richness, Shannon Index, *F/B* ratio, *Fusobacteria* Abundance, and Plasma LBP Levels, Directly Comparing Patients with Matched Household Controls. Statistics: For contrasts between patients and matched pair controls, Wilcoxon matched-pairs signed rank test was utilized. *P<0.05, **P<0.01, ***P<0.001, ****P<0.0001.

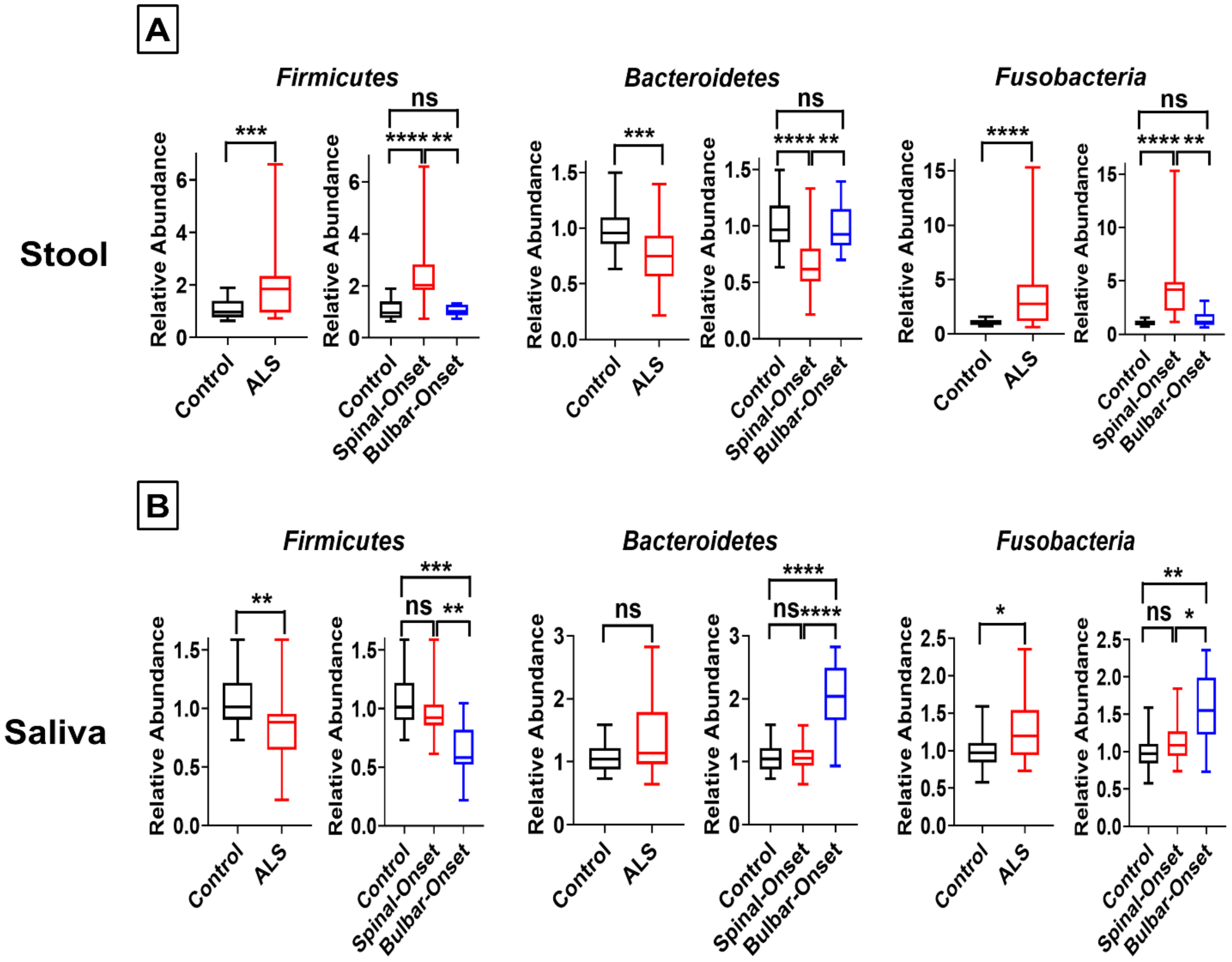

Supplemental Figure 4. Verification of Major Gut- and Oral- Microbiome Changes Using qPCR. (A) Relative abundance in the gut microbiome of *Firmicutes*, *Bacteroidetes*, and *Fusobacteria*. Results represent both ALS (spinal- and bulbar-onset ALS combined) or spinal- and bulbar-onset ALS quantified separately. **(B)** Relative abundance in the oral microbiome of *Firmicutes*, *Bacteroidetes*, and *Fusobacteria*. Results represent both ALS (spinal- and bulbar-onset ALS combined) or spinal- and bulbar-onset ALS quantified separately. **Statistics:** For comparisons between control and ALS patients (combined), Mann-Whitney test was utilized. For contrasts between control, spinal-onset ALS, and bulbar-onset ALS, Kruskal-Wallis test with Dunn's post-hoc multiple comparisons test was utilized. Values represent sample median with interquartile range. *P<0.05, **P<0.01, ***P<0.001, ****P<0.0001.

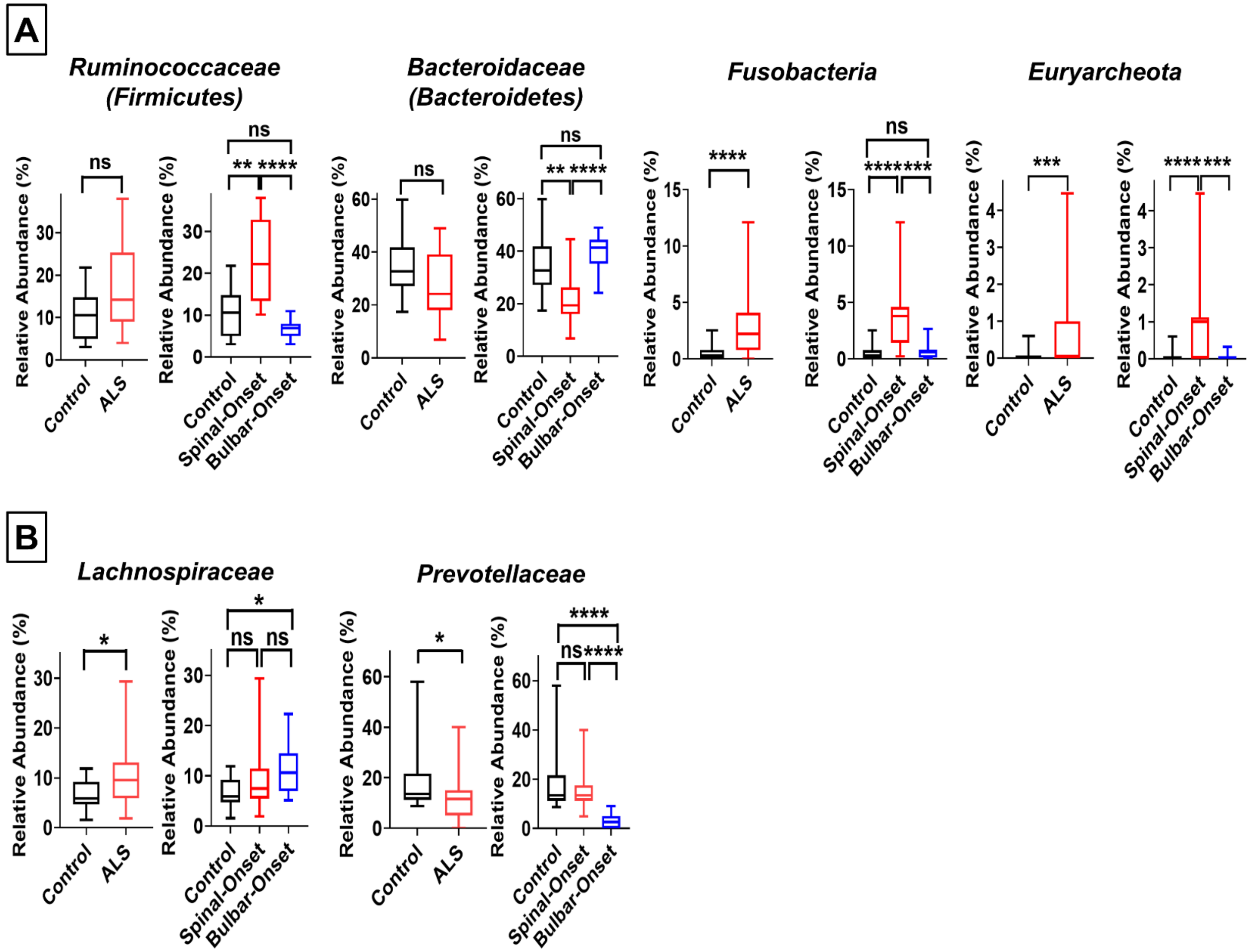

Supplemental Figure 5. Gut Microbiome Affected in Only Spinal- or Bulbar-Onset ALS. (A) Percent abundance of *Ruminococcaceae* (a member of the *Firmicutes* phyla), *Bacteroidaceae* (a member of the *Bacteroidetes* phyla), *Fusobacteria*, and *Euryarcheota* in the gut microbiome. Results represent both ALS (spinal- and bulbar-onset ALS combined) or spinal- and bulbar-onset ALS quantified separately. **(B)** Percent abundance of *Lachnospiraceae* and *Prevotellaceae* in the gut microbiome. **Statistics:** For comparisons between control and ALS patients (combined), Mann-Whitney test was utilized. For contrasts between control, spinal-onset ALS, and bulbar-onset ALS, Kruskal-Wallis test with Dunn's post-hoc multiple comparisons test was utilized. Values represent sample median with interquartile range. *P<0.05, **P<0.01, ***P<0.001, ****P<0.0001.

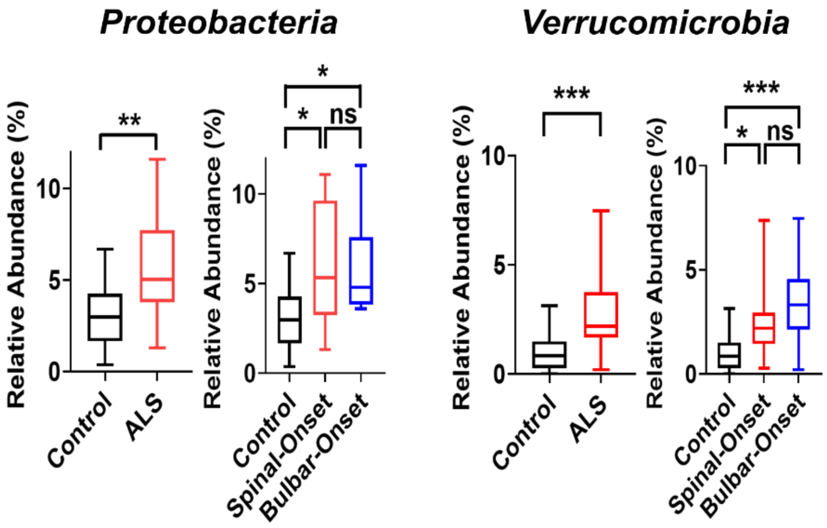

Supplemental Figure 6. Gut Microbiome Affected in Both Spinal- and Bulbar-Onset ALS. Percent abundance of *Proteobacteria* and *Verrucomicrobia* in the gut microbiome. Results represent both ALS (spinal- and bulbar-onset ALS combined) or spinal- and bulbar-onset ALS quantified separately. **Statistics:** For comparisons between control and ALS patients (combined), Mann-Whitney test was utilized. For contrasts between control, spinal-onset ALS, and bulbar-onset ALS, Kruskal-Wallis test with Dunn's post-hoc multiple comparisons test was utilized. Values represent sample median with interquartile range. *P<0.05, **P<0.01, ***P<0.001, ****P<0.0001.

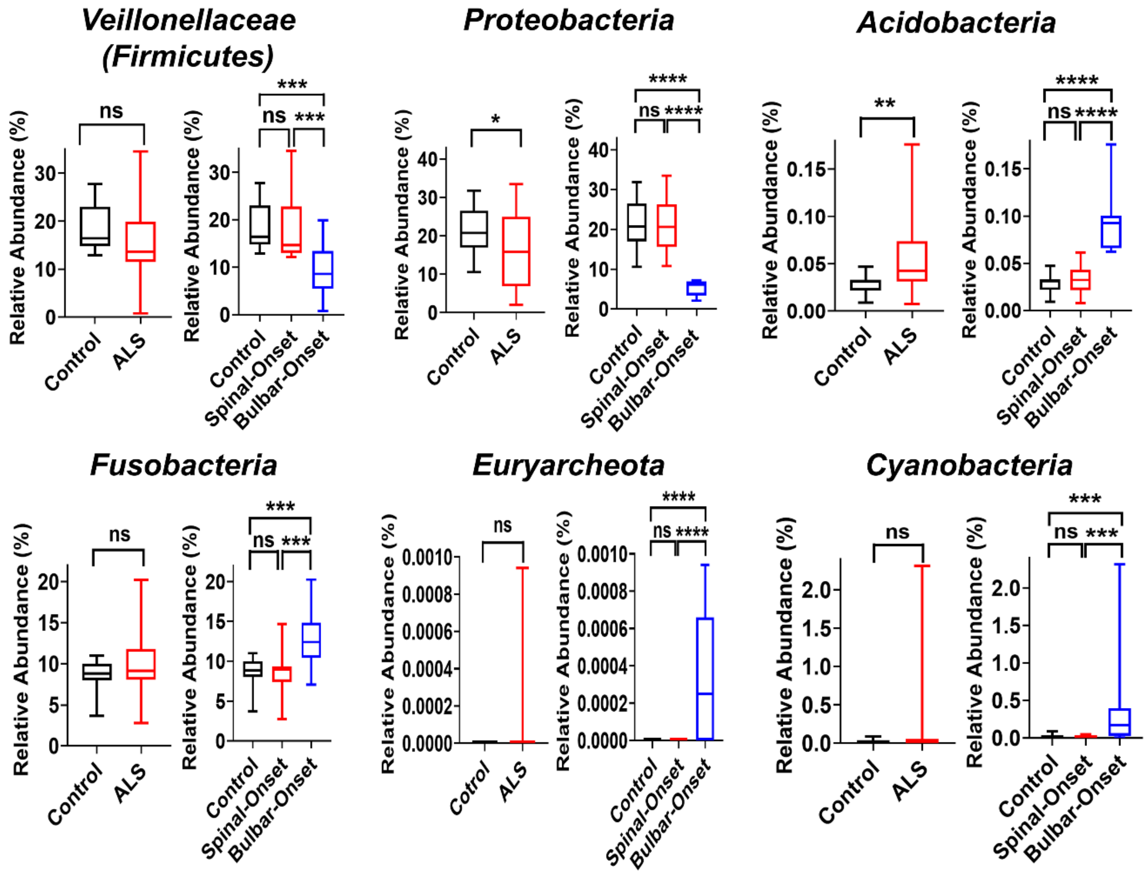

Supplemental Figure 7. Oral Microbiome Affected in Only Bulbar-Onset ALS. Percent abundance of *Veillonellaceae* (a member of the *Firmicutes* phyla), *Proteobacteria*, *Acidobacteria*, *Fusobacteria*, *Euryarcheota*, and *Cyanobacteria* in the oral microbiome. Results represent both ALS (spinal- and bulbar-onset ALS combined) or spinal- and bulbar-onset ALS quantified separately. **Statistics:** For comparisons between control and ALS patients (combined), Mann-Whitney test was utilized. For contrasts between control, spinal-onset ALS, and bulbar-onset ALS, Kruskal-Wallis test with Dunn's post-hoc multiple comparisons test was utilized. Values represent sample median with interquartile range. **P*<0.05, ***P*<0.01, ****P*<0.001, *****P*<0.0001.

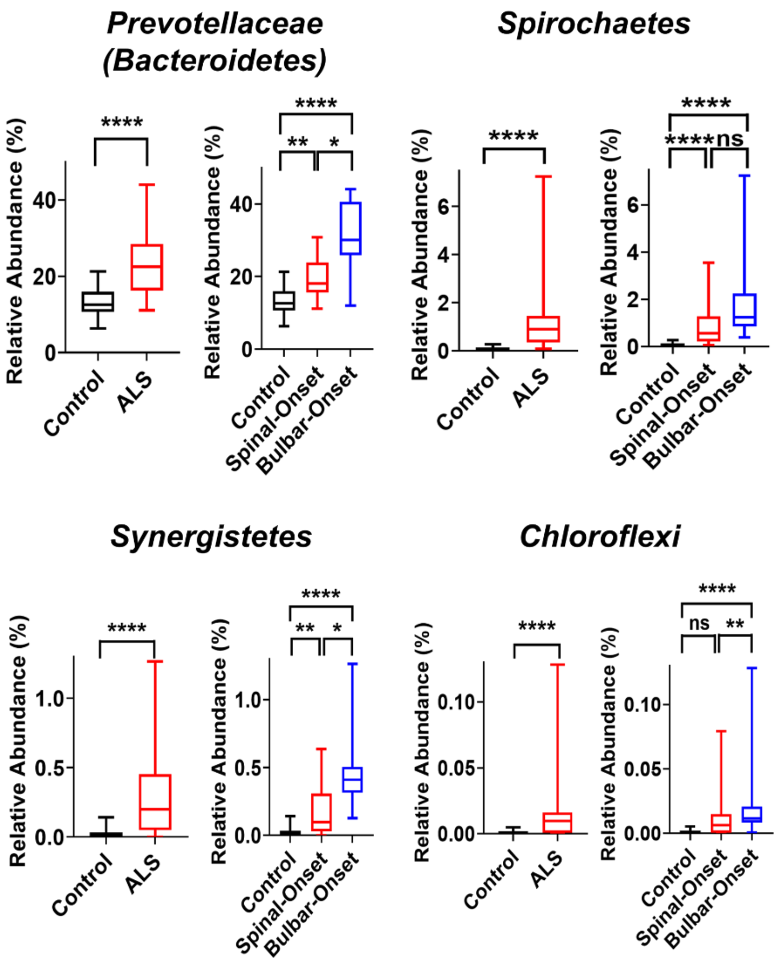

Supplemental Figure 8. Oral Microbiome Affected in Both Spinal- and Bulbar-Onset ALS. Percent abundance of *Prevotellaceae* (a member of the *Bacteroidetes* phyla), *Spirochaetes*, *Synergistetes*, and *Chloroflexi* in the oral microbiome. Results represent both ALS (spinal- and bulbar-onset ALS combined) or spinal- and bulbar-onset ALS quantified separately. **Statistics:** For comparisons between control and ALS patients (combined), Mann-Whitney test was utilized. For contrasts between control, spinal-onset ALS, and bulbar-onset ALS, Kruskal-Wallis test with Dunn's post-hoc multiple comparisons test was utilized. Values represent sample median with interquartile range. *P<0.05, **P<0.01, ***P<0.001, ****P<0.0001.

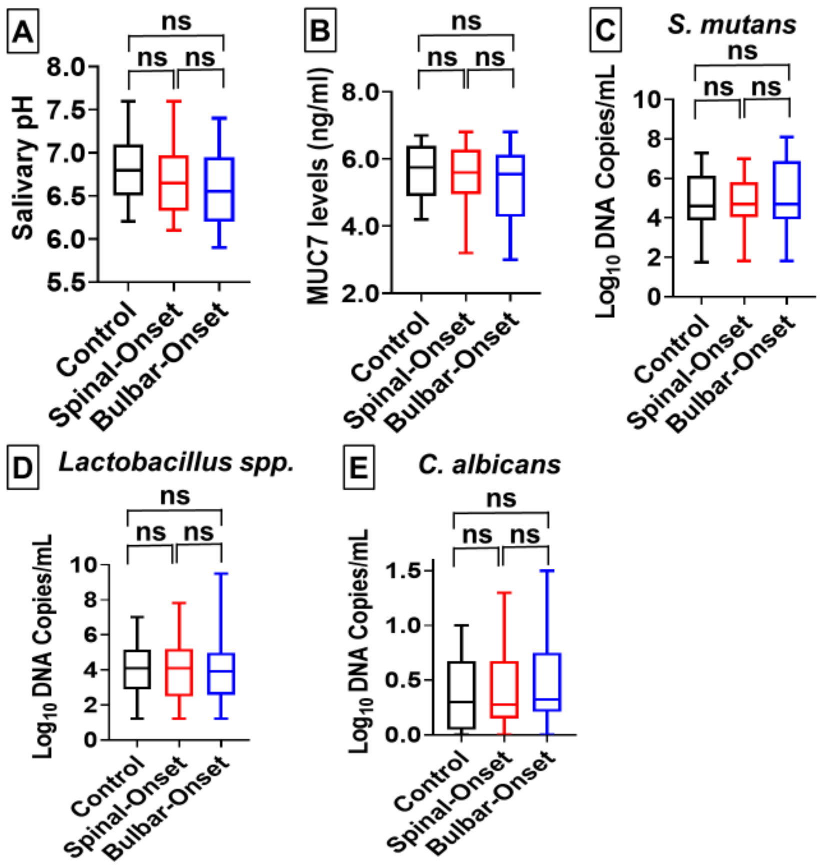

Supplemental Figure 9. Oral Health and Markers of Caries in Controls and ALS Patients (A) Salivary pH **(B)** Salivary MUC7 levels **(C)** Salivary abundance of *Streptococcus mutans* **(D)** Salivary abundance of *Lactobacillus spp.* **(E)** Salivary abundance of *Candida albicans*. **Statistics:** For contrasts between control, spinal-onset ALS, and bulbar-onset ALS, Kruskal-Wallis test with Dunn's post-hoc multiple comparisons test was utilized. *P<0.05, **P<0.01, ***P<0.001, ****P<0.0001.

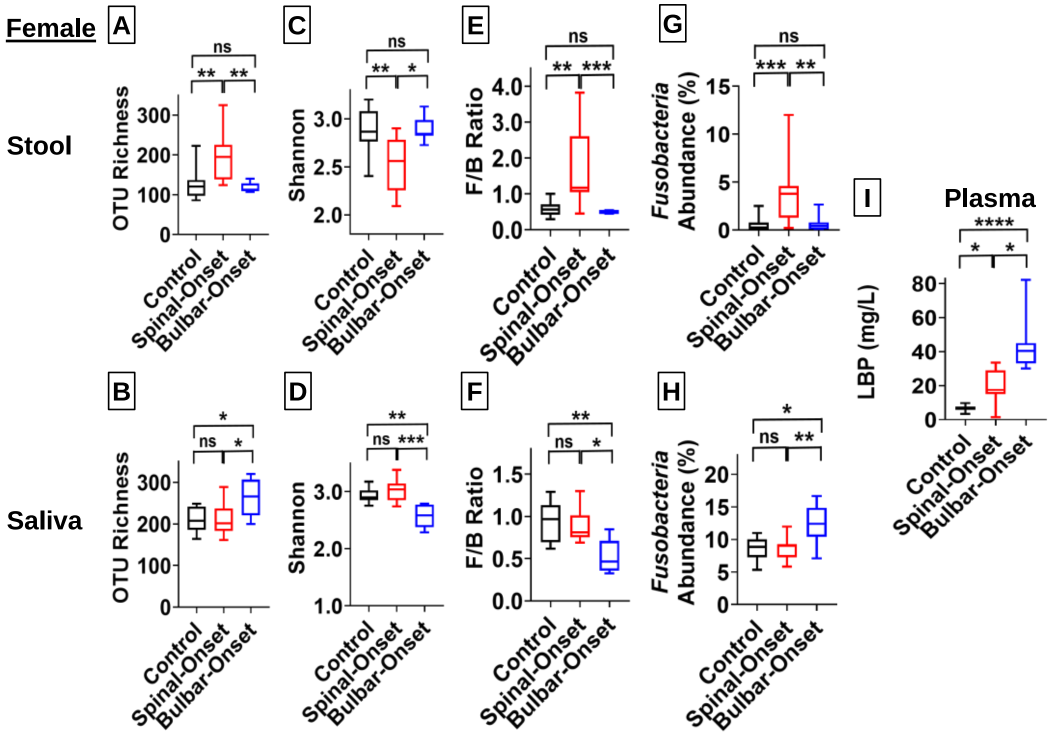

Supplemental Figure 10. OTU Richness, Shannon Index, *F*/*B* Ratio, *Fusobacteria* Abundance, and Plasma LBP Levels in Females Only. Statistics: For contrasts between control, spinal-onset ALS, and bulbar-onset ALS, Kruskal-Wallis test with Dunn's post-hoc multiple comparisons test was utilized. **P*<0.05, ***P*<0.01, ****P*<0.001, *****P*<0.0001.

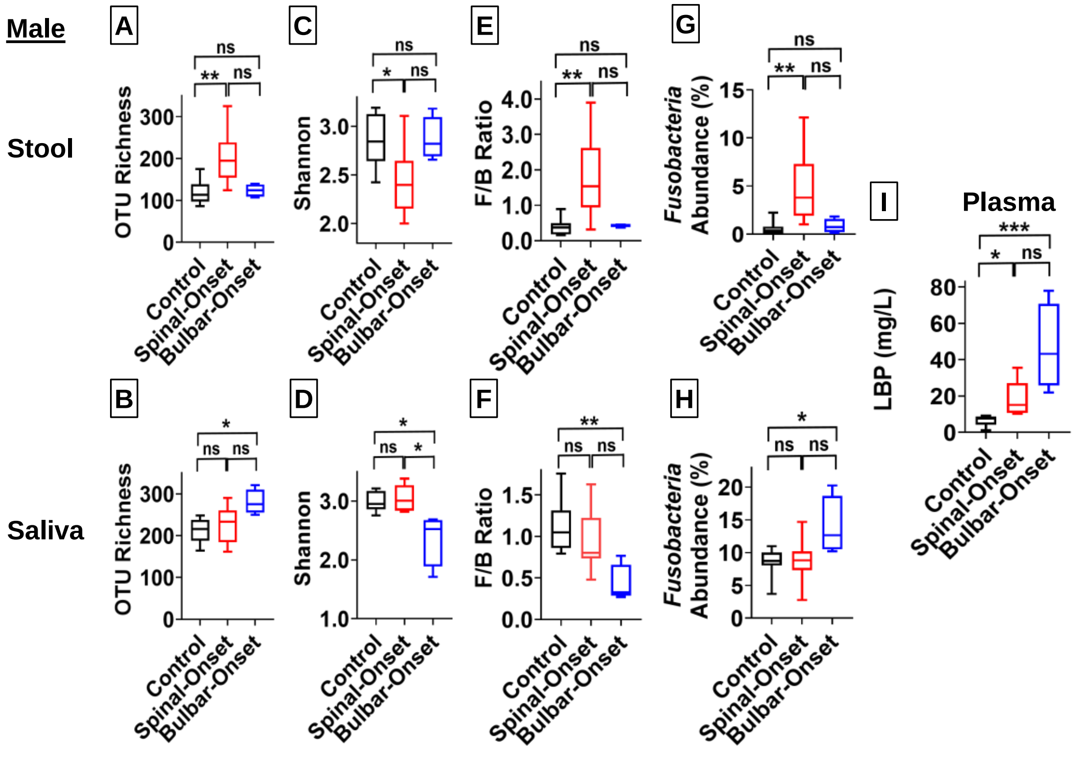

Supplemental Figure 11. OTU Richness, Shannon Index, *F*/*B* Ratio, *Fusobacteria* Abundance, and Plasma LBP Levels in Males Only. Statistics: For contrasts between control, spinal-onset ALS, and bulbar-onset ALS, Kruskal-Wallis test with Dunn's post-hoc multiple comparisons test was utilized. **P*<0.05, ***P*<0.01, ****P*<0.001, *****P*<0.0001.

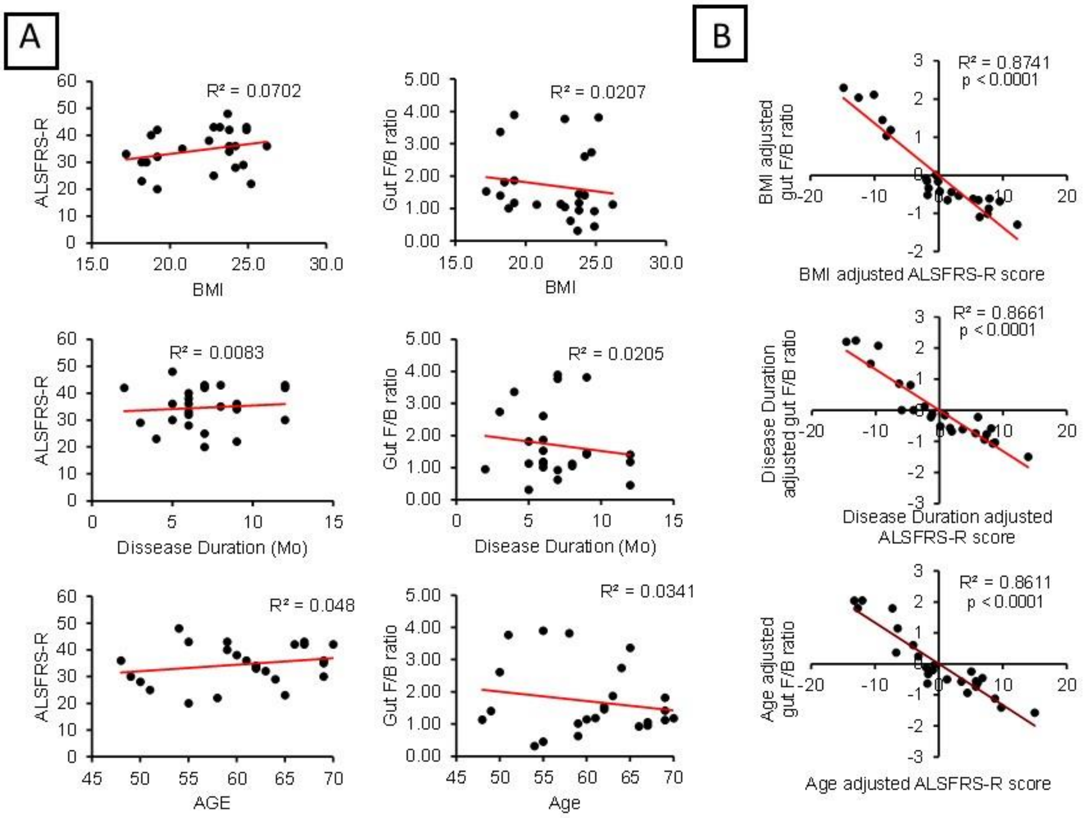

Supplemental Figure 12. Plot of Spinal-Onset ALS Patient ALSFRS-R Scores against Gut *F*/*B* Ratio Adjusted by BMI, Disease Duration, and Age (A) Spinal-onset ALS patient ALSFRS-R scores and gut *F*/*B* ratio are plotted against patient age, BMI, and time since disease diagnosis. **(B)** Residuals from plots in (A) are shown to allow evaluation of correlation between gut *F*/*B* ratio and spinal-onset patient ALSFRS-R scores having accounted for variation due to BMI (top), Disease duration (middle), Age (bottom). **Statistics:** Significance is based on simple linear regression analysis.

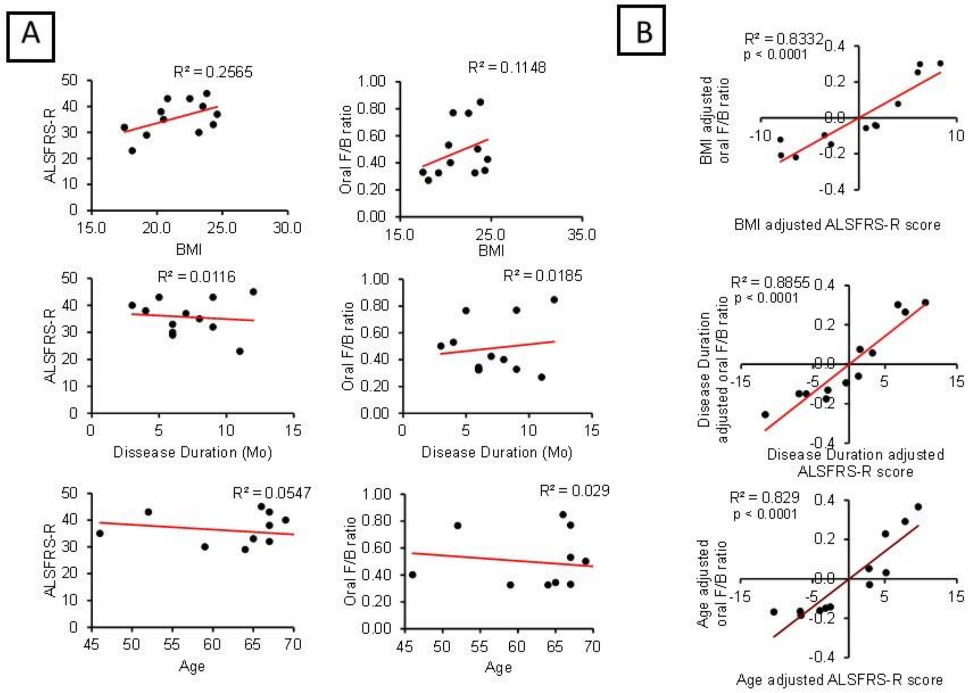

Supplemental Figure 13. Plot of Bulbar-Onset ALS Patient ALSFRS-R Scores Against Oral *F/B* Ratio Adjusted by BMI, Disease Duration, and Age (A) Bulbar-onset ALS patient ALSFRS-R scores and oral *F/B* ratio are plotted against patient age, BMI, and time since disease diagnosis. **(B)** Residuals from plots in A are shown to allow evaluation of correlation between oral *F/B* ratio and spinal patient ALSFRS-R scores having accounted for variation due to BMI (top), Disease duration (middle), Age (bottom). **Statistics:** Significance is based on simple linear regression analysis.

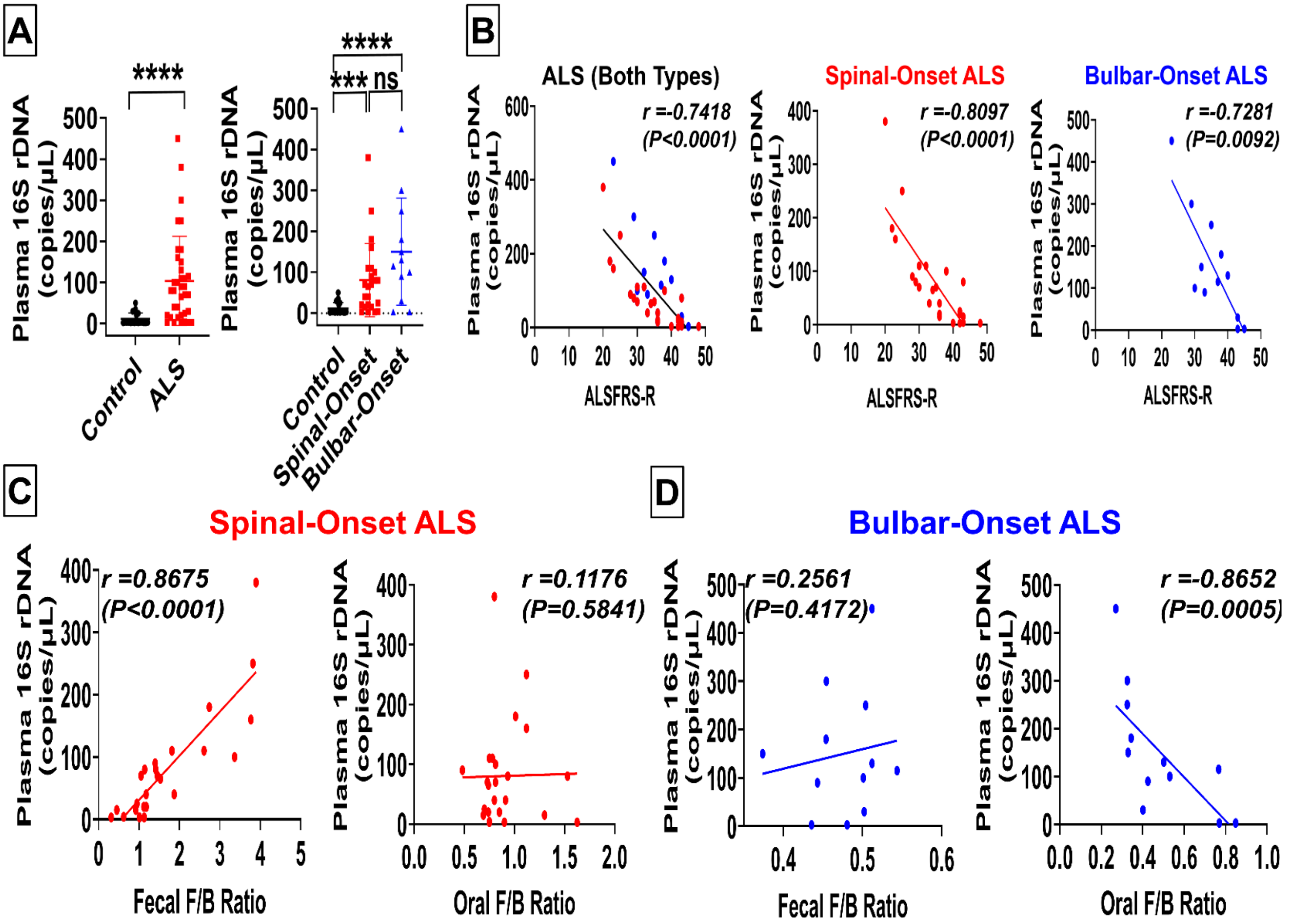

Supplemental Figure 14. Blood Microbial Content and Its Association with ALS Disease Severity and Microbial Dysbiosis. (A) qPCR measures of 16S rDNA in all ALS patients (left) and patients divided into spinal-onset ALS and bulbar-onset ALS patients (right). Values are shown as copy numbers per µl derived from blood plasma. **(B)** Linear regression analysis comparing patient plasma 16S rDNA and symptom severity (ALSFRS-R score) for all ALS patients combined (left), spinal-onset ALS patients only (middle), and bulbar-onset ALS patients only (right). Spinal-onset ALS patients shown in red, bulbar-onset ALS patients shown in blue. In both spinal- and bulbar-onset ALS patients, higher 16S rDNA levels (greater microbial translocation to the blood) were strongly associated with lower ALSFRS-R score (greater ALS severity). **(C)** Linear regression analyses comparing fecal *F/B* ratio and plasma 16S rDNA levels (left) and oral *F/B* ratio and plasma 16S rDNA levels (right) in spinal-onset ALS patients. In spinal-onset ALS patients, fecal *F/B* ratio showed strong correlations with plasma 16S rDNA levels, but oral *F/B* ratio showed poor correlations with plasma 16S rDNA levels. In spinal-onset ALS patients, higher fecal *F/B* ratio (greater gut-dysbiosis) was strongly associated with greater plasma 16S rDNA levels (greater microbial translocation to the blood). **(D)** Linear regression analyses comparing fecal *F/B* ratio and plasma 16S rDNA levels (left) and oral *F/B* ratio and plasma 16S rDNA levels (right) in bulbar-onset ALS patients. In bulbar-onset ALS patients, oral *F/B* ratio showed strong correlations with plasma 16S rDNA levels, but fecal *F/B* ratio showed poor correlations with plasma 16S rDNA levels. In bulbar-onset ALS patients, lower oral *F/B* ratio (greater oral-dysbiosis) was strongly associated with greater plasma 16S rDNA levels (greater microbial translocation to the blood). **Statistics:** For comparisons between control and ALS patients (combined), Mann-Whitney test was utilized. For contrasts between control, spinal-onset ALS, and bulbar-onset ALS, Kruskal-Wallis test with Dunn's post-hoc multiple comparison test was utilized. **P*<0.05, ***P*<0.01, ****P*<0.001, *****P*<0.0001. For correlations between 16S rDNA & ALSFRS-R and 16S rDNA & fecal/oral *F/B* ratio, statistical significance was established using linear regression analysis and Spearman's correlation coefficient (*r*) test.

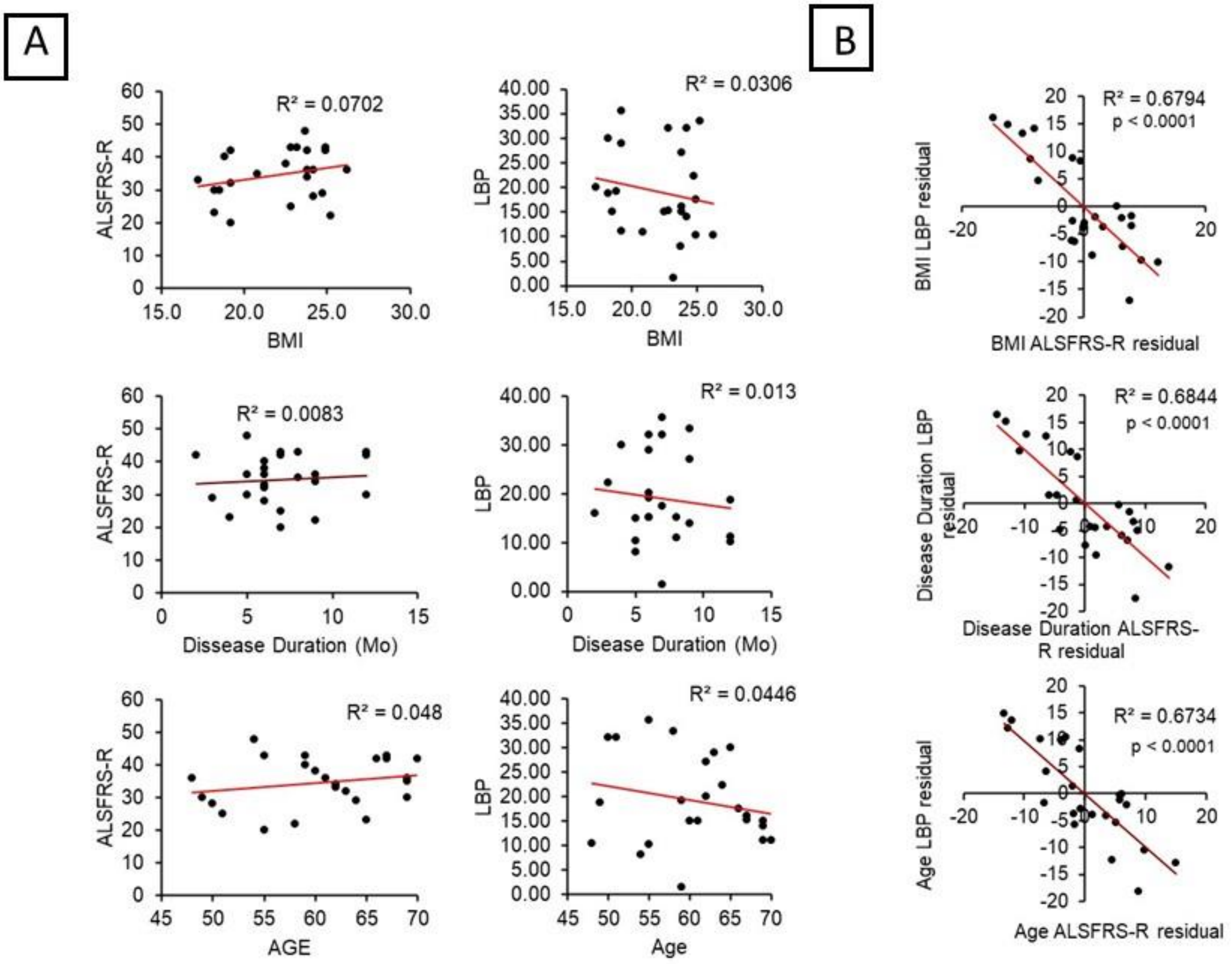

Supplemental Figure 15. Plot of Spinal-Onset ALS Patient ALSFRS-R Scores Against Plasma LBP Adjusted by BMI, Disease Duration, and Age. **(A)** Spinal-onset ALS patient ALSFRS-R scores and plasma LBP are plotted against patient age, BMI, and time since disease diagnosis. **(B)** Residuals from plots in (A) are shown to allow evaluation of correlation between plasma LBP and spinal-onset patient ALSFRS-R scores having accounted for variation due to BMI (top), Disease duration (middle), Age (bottom). **Statistics:** Significance is based on simple linear regression analysis.

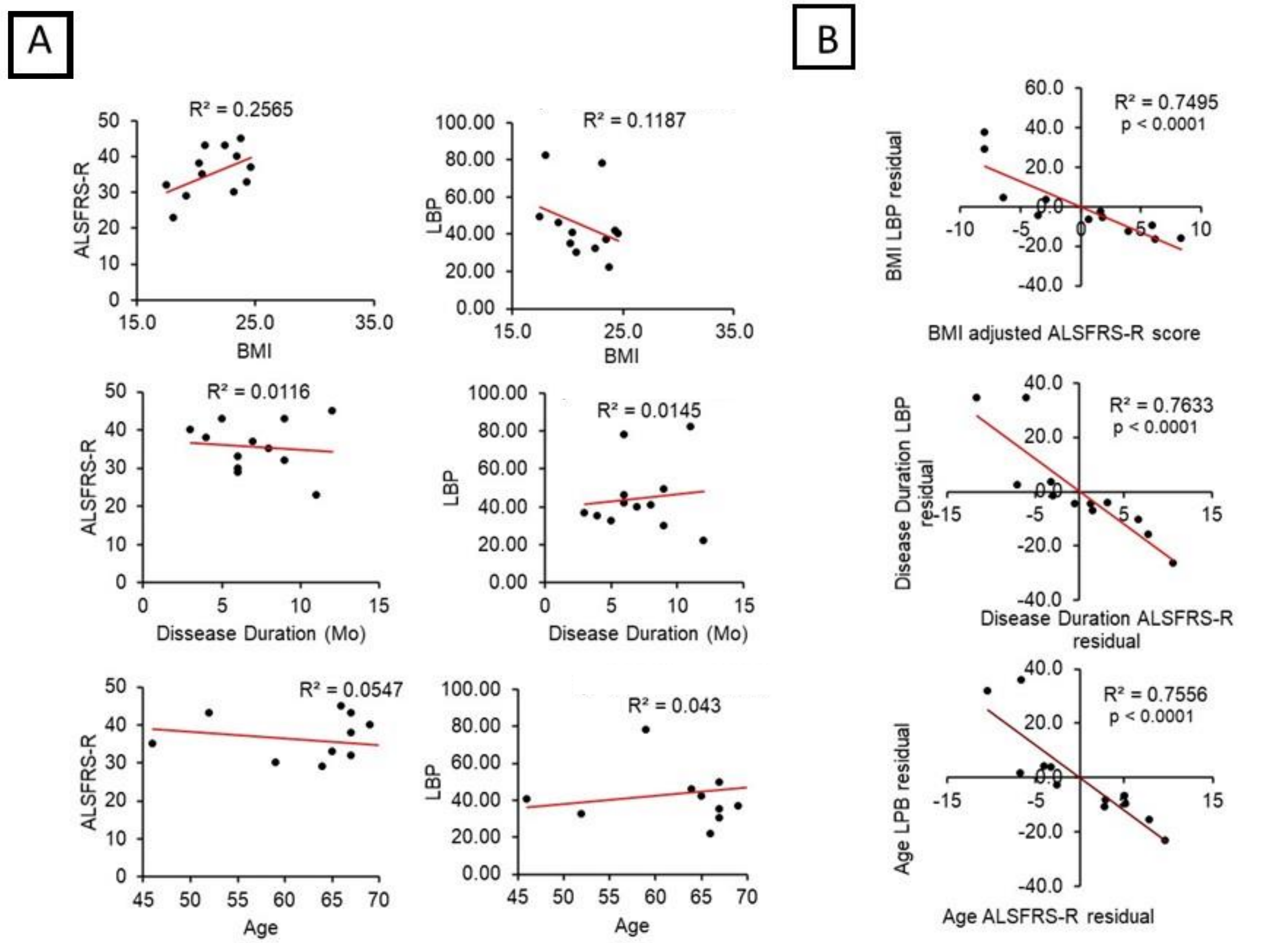

Supplemental Figure 16. Plot of Bulbar-Onset ALS Patient ALSFRS-R Scores Against Plasma LBP Adjusted by BMI, Disease Duration, and Age. (A) Bulbar-onset ALS patient ALSFRS-R scores and plasma LBP are plotted against patient age, BMI, and time since disease diagnosis. **(B)** Residuals from plots in (A) are shown to allow evaluation of correlation between plasma LBP and bulbar patient ALSFRS-R scores having accounted for variation due to BMI (top), Disease duration (middle), Age (bottom). **Statistics:** Significance is based on simple linear regression analysis.

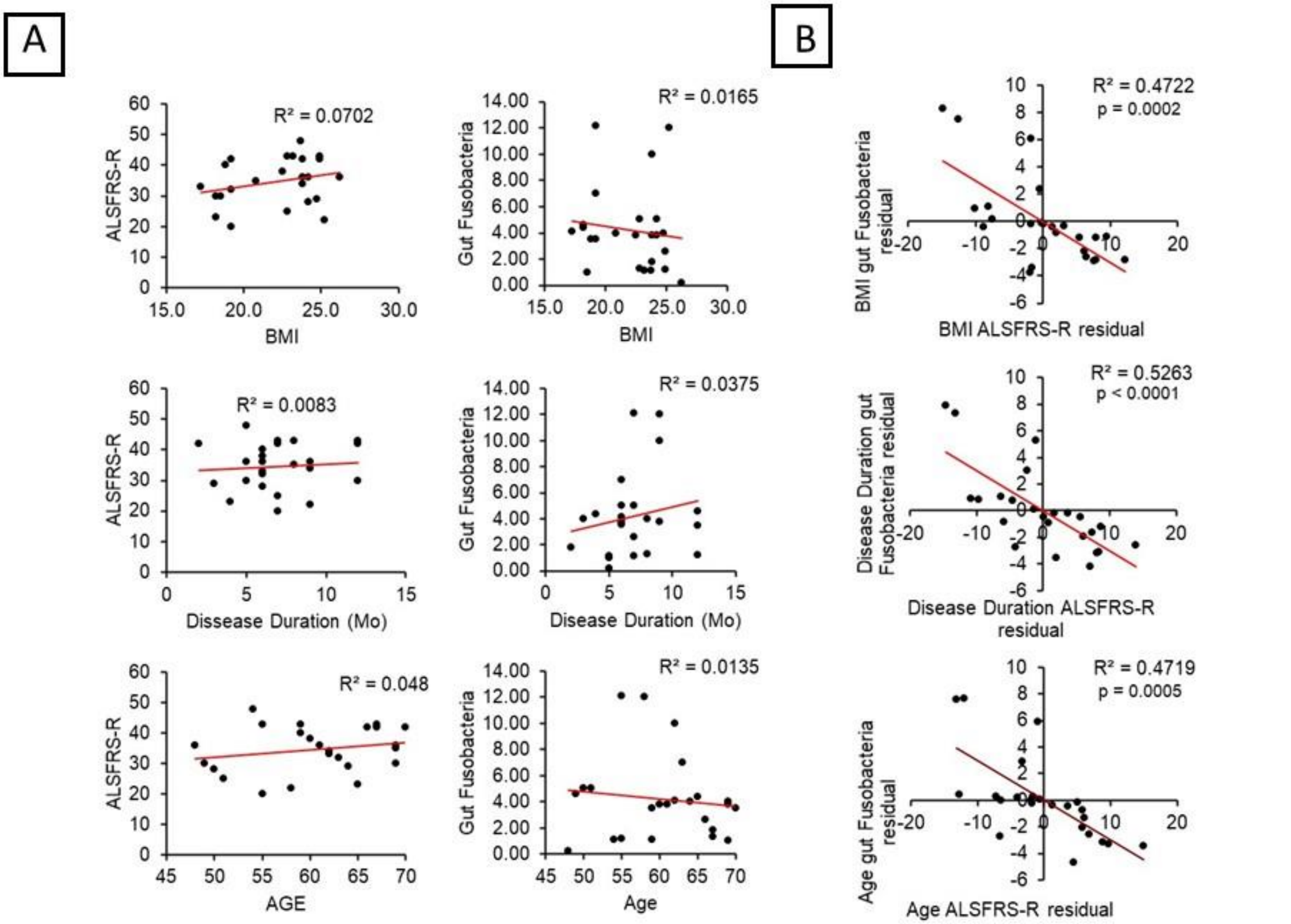

Supplemental Figure 17. Plot of Spinal-Onset ALS Patient ALSFRS-R Scores Against Gut *Fusobacteria* Adjusted by BMI, Disease Duration, and Age. (A) Spinal-onset ALS patient ALSFRS-R scores and gut *Fusobacteria* are plotted against patient age, BMI, and time since disease diagnosis. **(B)** Residuals from plots in (A) are shown to allow evaluation of correlation between gut *Fusobacteria* and spinal-onset patient ALSFRS-R scores having accounted for variation due to BMI (top), Disease duration (middle), Age (bottom). **Statistics:** Significance is based on simple linear regression analysis.

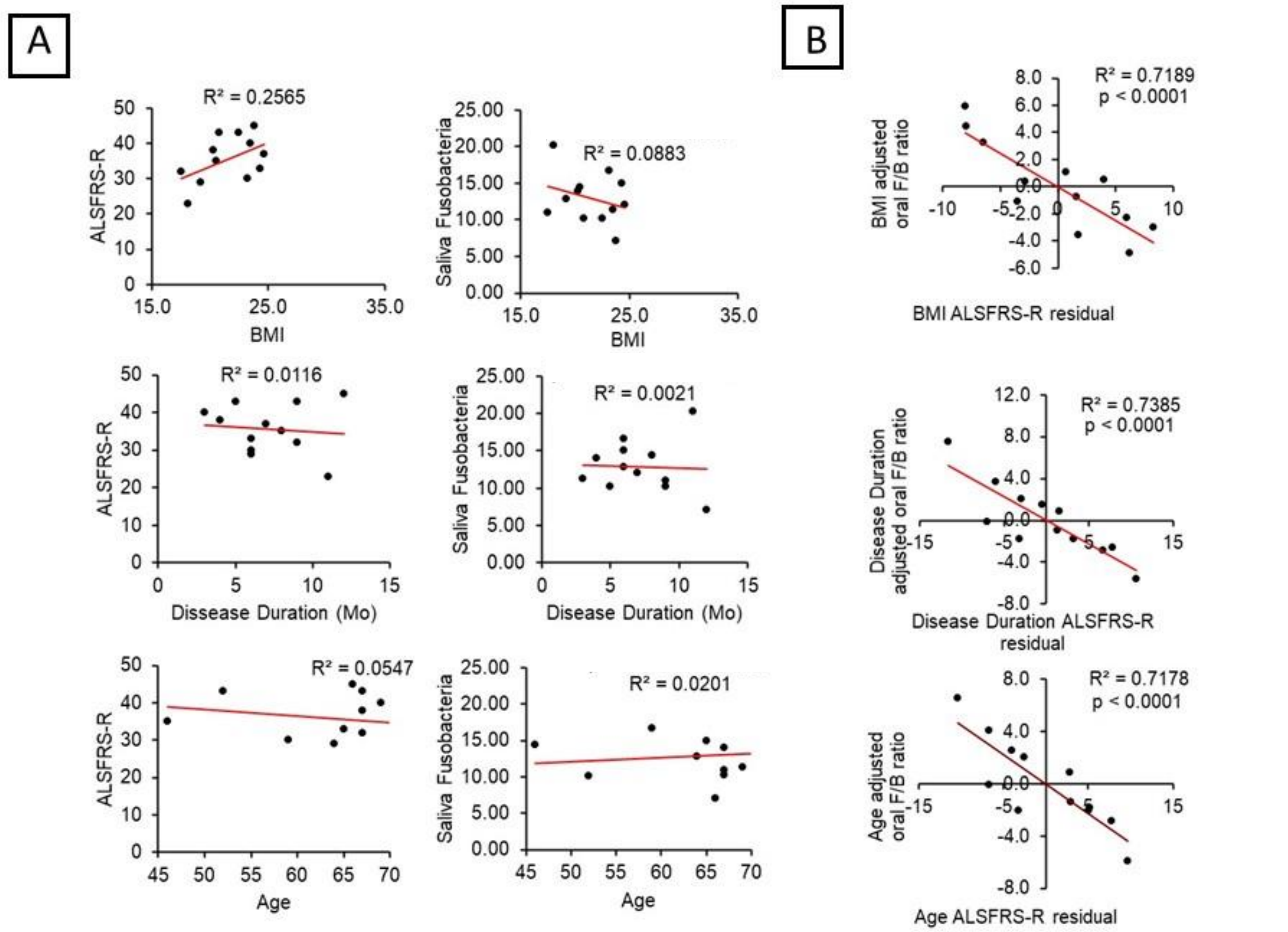

Supplemental Figure 18. Plot of Bulbar-Onset ALS Patient ALSFRS-R Scores Against Oral *Fusobacteria* Adjusted by BMI, Disease Duration, and Age. (A) Bulbar-onset ALS patient ALSFRS-R scores and oral *Fusobacteria* are plotted against patient age, BMI, and time since disease diagnosis. **(B)** Residuals from plots in (A) are shown to allow evaluation of correlation between oral *Fusobacteria* and bulbar patient ALSFRS-R scores having accounted for variation due to BMI (top), Disease duration (middle), Age (bottom). **Statistics:** Significance is based on simple linear regression analysis.

Stool

Sample	% of reads passing at least Q_{20} (reads below Q_{20} were excluded)	% of reads classified to an identified taxon
Control 1	96.24%	90.42%
Control 2	92.17%	90.38%
Control 3	96.79%	95.38%
Control 4	98.50%	97.80%
Control 5	97.44%	94.39%
Control 6	96.07%	90.92%
Control 7	94.59%	92.68%
Control 8	97.60%	95.39%
Control 9	100.00%	100.00%
Control 10	100.00%	99.90%
Control 11	100.00%	99.70%
Control 12	100.00%	100.00%
Control 13	100.00%	99.80%
Control 14	100.00%	99.90%
Control 15	100.00%	99.80%
Control 16	100.00%	100.00%
Control 17	100.00%	99.90%
Control 18	98.25%	97.10%
Control 19	95.60%	93.80%
Control 20	98.42%	96.50%
Spinal-Onset ALS Patient 1	95.51%	90.57%
Spinal-Onset ALS Patient 2	95.77%	90.79%
Spinal-Onset ALS Patient 3	97.37%	90.94%
Spinal-Onset ALS Patient 4	96.22%	90.28%
Spinal-Onset ALS Patient 5	100.00%	99.90%
Spinal-Onset ALS Patient 6	98.25%	94.27%
Spinal-Onset ALS Patient 7	100.00%	99.80%
Spinal-Onset ALS Patient 8	99.60%	92.89%
Spinal-Onset ALS Patient 9	100.00%	100.00%
Spinal-Onset ALS Patient 10	99.20%	95.40%
Spinal-Onset ALS Patient 11	93.38%	90.96%
Spinal-Onset ALS Patient 12	96.41%	95.28%
Spinal-Onset ALS Patient 13	97.05%	95.50%
Spinal-Onset ALS Patient 14	96.52%	92.45%
Spinal-Onset ALS Patient 15	95.71%	93.79%
Spinal-Onset ALS Patient 16	100.00%	99.90%
Spinal-Onset ALS Patient 17	100.00%	99.90%
Spinal-Onset ALS Patient 18	100.00%	99.90%
Spinal-Onset ALS Patient 19	100.00%	100.00%
Spinal-Onset ALS Patient 20	100.00%	99.80%
Spinal-Onset ALS Patient 21	100.00%	99.80%
Spinal-Onset ALS Patient 22	100.00%	100.00%
Spinal-Onset ALS Patient 23	100.00%	98.90%
Spinal-Onset ALS Patient 24	100.00%	97.80%
Bulbar-Onset ALS Patient 1	93.12%	90.55%
Bulbar-Onset ALS Patient 2	92.93%	90.30%
Bulbar-Onset ALS Patient 3	95.66%	92.39%
Bulbar-Onset ALS Patient 4	96.24%	91.07%
Bulbar-Onset ALS Patient 5	95.57%	93.52%
Bulbar-Onset ALS Patient 6	100.00%	99.90%
Bulbar-Onset ALS Patient 7	100.00%	100.00%
Bulbar-Onset ALS Patient 8	100.00%	99.90%
Bulbar-Onset ALS Patient 9	100.00%	99.90%
Bulbar-Onset ALS Patient 10	100.00%	99.90%
Bulbar-Onset ALS Patient 11	100.00%	100.00%
Bulbar-Onset ALS Patient 12	100.00%	100.00%

Saliva

Sample	% of reads passing at least Q_{20} (reads below Q_{20} were excluded)	% of reads classified to an identified taxon
Control 1	96.37%	92.89%
Control 2	95.42%	94.83%
Control 3	95.74%	94.55%
Control 4	96.21%	95.42%
Control 5	96.16%	95.25%
Control 6	96.37%	94.33%
Control 7	95.49%	94.28%
Control 8	100.00%	100.00%
Control 9	100.00%	99.90%
Control 10	100.00%	99.90%
Control 11	100.00%	98.80%
Control 12	100.00%	99.90%
Control 13	100.00%	99.90%
Control 14	98.10%	97.25%
Control 15	100.00%	99.90%
Control 16	99.25%	98.90%
Control 17	100.00%	99.90%
Control 18	98.45%	97.52%
Control 19	100.00%	99.90%
Control 20	100.00%	99.90%
Spinal-Onset ALS Patient 1	95.10%	94.93%
Spinal-Onset ALS Patient 2	97.20%	96.50%
Spinal-Onset ALS Patient 3	95.16%	94.78%
Spinal-Onset ALS Patient 4	95.46%	94.51%
Spinal-Onset ALS Patient 5	100.00%	99.90%
Spinal-Onset ALS Patient 6	100.00%	99.90%
Spinal-Onset ALS Patient 7	99.82%	98.50%
Spinal-Onset ALS Patient 8	98.25%	97.20%
Spinal-Onset ALS Patient 9	99.20%	98.35%
Spinal-Onset ALS Patient 10	95.90%	93.30%
Spinal-Onset ALS Patient 11	96.37%	94.33%
Spinal-Onset ALS Patient 12	95.45%	94.29%
Spinal-Onset ALS Patient 13	96.10%	91.84%
Spinal-Onset ALS Patient 14	95.98%	94.12%
Spinal-Onset ALS Patient 15	94.68%	93.60%
Spinal-Onset ALS Patient 16	100.00%	99.30%
Spinal-Onset ALS Patient 17	100.00%	99.90%
Spinal-Onset ALS Patient 18	100.00%	99.80%
Spinal-Onset ALS Patient 19	100.00%	99.80%
Spinal-Onset ALS Patient 20	100.00%	99.80%
Spinal-Onset ALS Patient 21	100.00%	99.90%
Spinal-Onset ALS Patient 22	100.00%	100.00%
Spinal-Onset ALS Patient 23	100.00%	99.90%
Spinal-Onset ALS Patient 24	100.00%	99.90%
Bulbar-Onset ALS Patient 1	96.86%	96.16%
Bulbar-Onset ALS Patient 2	100.00%	99.90%
Bulbar-Onset ALS Patient 3	95.77%	94.23%
Bulbar-Onset ALS Patient 4	99.80%	98.90%
Bulbar-Onset ALS Patient 5	96.34%	95.67%
Bulbar-Onset ALS Patient 6	97.30%	95.78%
Bulbar-Onset ALS Patient 7	100.00%	99.90%
Bulbar-Onset ALS Patient 8	100.00%	99.10%
Bulbar-Onset ALS Patient 9	100.00%	99.80%
Bulbar-Onset ALS Patient 10	100.00%	99.90%
Bulbar-Onset ALS Patient 11	100.00%	99.80%
Bulbar-Onset ALS Patient 12	100.00%	98.90%

Supplemental Figure 19. 16S read quality. Q_{20} (equivalent to > 1% error rate) represented the minimum quality criteria for inclusion in analysis. All reads below Q_{20} were excluded. The percentage of reads which could be classified to particular bacteria is also included.

Power analysis for OTU richness in stool

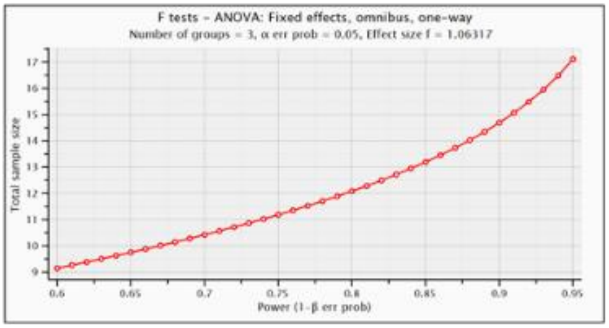

Power analysis for Shannon index in stool

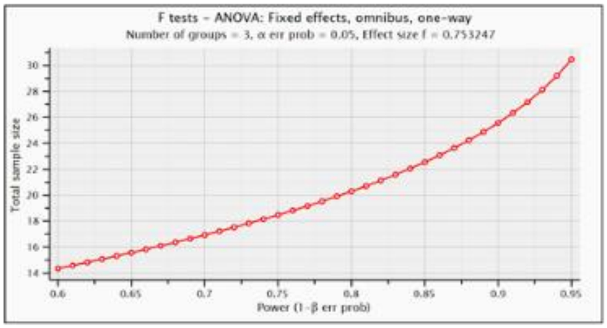

Power analysis for OTU richness in saliva

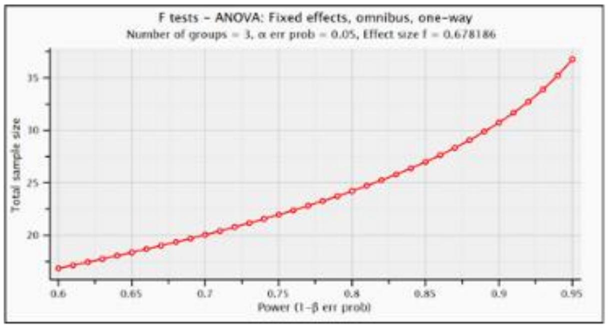

Power analysis for Shannon index in saliva

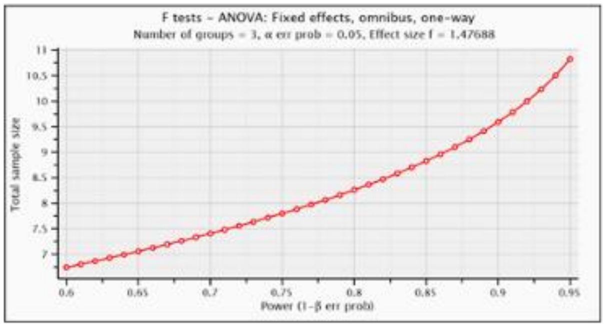

Power analysis for Bacteroidetes in stool

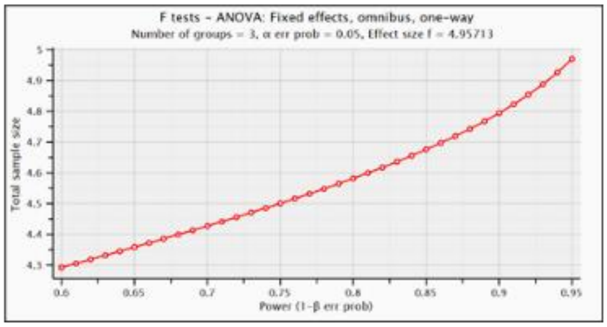

Power analysis for Firmicutes in stool

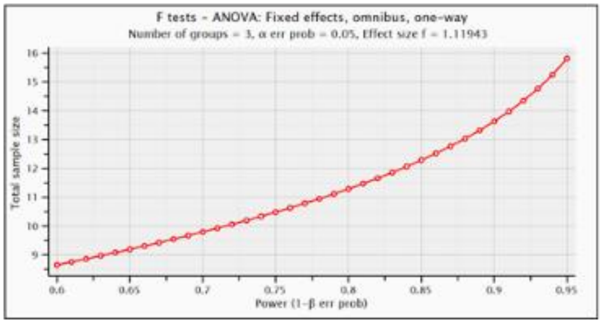

Power analysis for Bacteroidetes in saliva

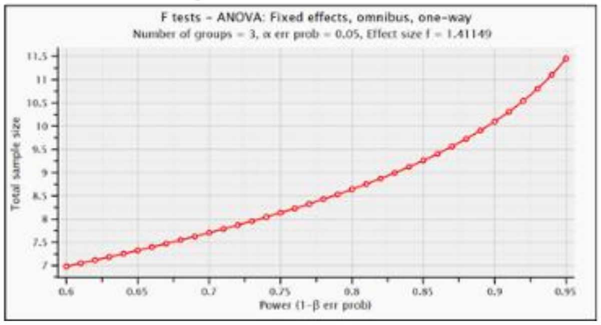

Power analysis for Firmicutes in saliva

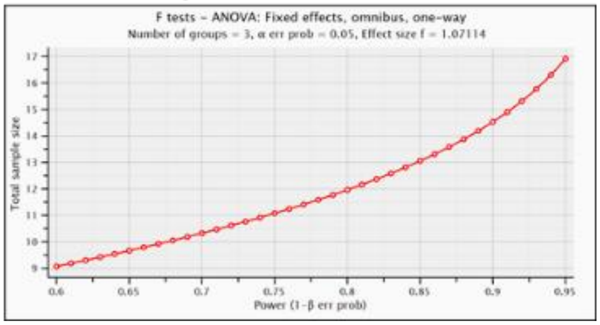

Supplemental Figure 20. Post-hoc Power Analysis for Key Conclusions Drawn in the Manuscript.
